# Proliferative Effect of Aqueous Extract of Sea Cucumber (*Holothuria parva*) Body Wall on Human Umbilical Cord Mesenchymal Stromal/Stem Cells

**DOI:** 10.3390/md21050267

**Published:** 2023-04-26

**Authors:** Poorya Rasekh, Ali Kameli, Arezoo Khoradmehr, Neda Baghban, Gholamhossein Mohebbi, Alireza Barmak, Iraj Nabipour, Hossein Azari, Yaser Heidari, Adel Daneshi, Afshar Bargahi, Zahra Khodabandeh, Shahrokh Zare, Alireza Afshar, Reza Shirazi, Sahar Almasi-Turk, Amin Tamadon

**Affiliations:** 1 The Persian Gulf Marine Biotechnology Research Center, The Persian Gulf Biomedical Sciences Research Institute, Bushehr University of Medical Sciences, Bushehr 7514633196, Iran; 2 Food Lab, Bushehr University of Medical Sciences, Bushehr 7518759577, Iran; 3 Stem Cells Technology Research Center, Shiraz University of Medical Sciences, Shiraz 71348-14336, Iran; 4 Department of Anatomy, School of Medical Sciences, Medicine, UNSW Sydney, Sydney 3052, Australia; 5 Department of Anatomical Sciences, School of Medicine, Bushehr University of Medical Sciences, Bushehr 7514633196, Iran; 6 PerciaVista R&D Co., Shiraz 7167683745, Iran; 7 Department for Scientific Work, West Kazakhstan Marat Ospanov Medical University, Aktobe 030012, Kazakhstan

**Keywords:** stem cells, sea cucumbers, proliferation, aqueous extract, bioactive compounds, epidermal growth factor

## Abstract

Sea cucumber extracts and their bioactive compounds have the potential for stem cell proliferation induction and for their beneficial therapeutic properties. In this study, human umbilical cord mesenchymal stromal/stem cells (hUC-MSCs) were exposed to an aqueous extract of *Holothuria parva* body walls. Proliferative molecules were detected using gas chromatography-mass spectrometry (GC-MS) analysis in an aqueous extract of *H. parva*. The aqueous extract concentrations of 5, 10, 20, 40, and 80 µg/mL and 10 and 20 ng/mL of human epidermal growth factor (EGF) as positive controls were treated on hUC-MSCs. MTT, cell count, viability, and cell cycle assays were performed. Using Western blot analysis, the effects of extracts of *H. parva* and EGF on cell proliferation markers were detected. Computational modeling was done to detect effective proliferative compounds in the aqueous extract of *H. parva*. A MTT assay showed that the 10, 20, and 40 µg/mL aqueous extract of *H. parva* had a proliferative effect on hUC-MSCs. The cell count, which was treated with a 20 µg/mL concentration, increased faster and higher than the control group (*p* < 0.05). This concentration of the extract did not have a significant effect on hUC-MSCs’ viability. The cell cycle assay of hUC-MSCs showed that the percentage of cells in the G2 stage of the extract was biologically higher than the control group. Expression of cyclin D1, cyclin D3, cyclin E, HIF-1α, and TERT was increased compared with the control group. Moreover, expression of p21 and PCNA decreased after treating hUC-MSCs with the extract. However, CDC-2/cdk-1 and ERK1/2 had almost the same expression as the control group. The expression of CDK-4 and CDK-6 decreased after treatment. Between the detected compounds, 1-methyl-4-(1-methyl phenyl)-benzene showed better affinity to CDK-4 and p21 than tetradecanoic acid. The *H. parva* aqueous extract showed proliferative potential on hUC-MSCs.

## 1. Introduction

Human umbilical cord mesenchymal stromal/stem cells (hUC-MSCs), as an allogeneic source of stem cells, have been clinically studied for skin repair [1]. hUC-MSCs can play roles in skin wound healing phases, including hemostasis, inflammation, proliferation, and maturation [2]. Skin injuries are categorized into superficial, partial-thickness, or full-thickness in terms of the damage caused to the skin structure [3]. These injuries can be treated by wound excision, skin grafting, skin substitutes, or wound dressings [3]. Introducing proliferative agents into skin wound dressings has the potential to improve tissue regeneration [4]. A combination of stem cell therapy and proliferative agents in complementary and alternative medicine can be promising for tissue regeneration [5,6].

Sea cucumbers have the ability of whole organ regeneration because they direct their wound healing abilities towards restoring their organs [7]. The bioactive compounds produced during wound healing and organ regeneration in sea cucumbers may allow us to repair human wounds. Sea cucumber extracts and their bioactive compounds have been studied for their beneficial therapeutic properties, including anti-cancer, anti-bacterial, anti-viral, anti-diabetic, and anticoagulant properties [8]. Recently, different types of extract from various species of sea cucumber have been shown to promote cell proliferation in cell lines and/or differentiation of pluripotent MSCs under standard in vitro or in vivo conditions [9,10,11,12]. Sea cucumber therapeutic effects have been gaining interest in complementary medicine because of their advantages over other semi-biological and synthetic proliferative inducers, such as availability, low toxicity, and comparatively low cost.

Compared to fibroblast growth factor (FGF), sea cucumber chondroitin sulfate caused the proliferation of endothelial cells [13]. Furthermore, sea cucumber-isolated sulfated polysaccharides increased the survival rate and proliferation of progenitor and stem cells in neural tissue [14]. In addition, it has a synergistic effect on growth with FGF, but this situation has not been seen with epidermal growth factor (EGF) [14]. The result is that these compounds can have a positive effect on the proliferation of nerve tissue progenitor cells [14], and they have also increased their ability of nerve tissue progenitor cells to migrate [15]. Growth factor effects of sea cucumber extract compounds in wound healing have been shown in the differentiation of MSCs into osteoblast cells [16] and as a dressing for diabetic wounds [17]. In wounds caused by alcohol [18] and drugs [19], the protective effects of sea cucumber compounds have been observed. The species of sea cucumber that is more abundant in the Persian Gulf is *Holothuria* spp. [20], from which compounds such as b-carotene, b-echinenone, canthaxanthin, phoenicoxanthin, astaxanthin, lutein, zeaxanthin, diatoxanthin, alloxanthin, and idoxanthin have been obtained [21].

We presume that bioactive molecules derived from sea cucumber could be utilized to enhance the proliferation of stem cells. In this project, we aimed to evaluate the impact of *Holothuria parva* aqueous extract on the viability and proliferation capacity of hUC-MSCs.

## 2. Results

### 2.1. Sea Cucumber Was Identified as H. parva 

*H. parva* is a small sea cucumber (<10 cm) with a spindle-shaped body thinned on both relatively thick extremities and integument; a ventral mouth surrounded by 20 small tentacles and a terminal anus. The identification of sea cucumbers was aided by the examination of the skeletal elements (ossicles) found in various parts of the body (Figure S1). Separated ossicles confirmed that the collected sea cucumbers were *H. parva* based on the identification keys [22,23].

### 2.2. Gas Chromatography-Mass Spectrometry (GC-MS) Analysis Detected Proliferative Compounds in the Watery Extract of H. parva

Sixty compounds were detected in the aqueous extract of *H. parva* via GC-MS analysis (Supplementary Material, Figure S2 and Table S1). As it was shown in Table S1, there were two molecules, including methyl ester octadecanoic acid [24,25,26] and 1-methyl-4-(1-methyl phenyl)-benzene [27,28,29] that have been proved to have proliferative biological activity. Other biological activities were also observed in other molecules of aqueous extract of *H. parva*, including anti-oxidant, anti-microbial, anti-inflammatory, etc. (Table S1).

### 2.3. Six Compounds Showed Better Affinity to Proteins

The docking process of each ligand-protein complex resulted in 10 conformations with different binding affinities as a docking score. Among them, the conformation with RMSD ≤2 Å and the lowest binding affinity, ΔG [U total in kcal/mol was chosen as the best or most stable one. The binding affinity values lower than 4 were assigned to good interactive relationships between the ligand and target. Table S2 presents ΔG corresponding to the best conformations. The binding energies are in the range of 2.5 to 7.9 Kcal/mol with a mean value of 4.7 Kcal/mol. The highest ΔG was related to the complex of p21 with decane 4-methyl- and tridecane. The lowest ΔG was related to the complex of cyclin D3 with 3,4-dihydro-1h-isoquinoline-2-carboxamidine hydrochloride.

**Figure 1:**
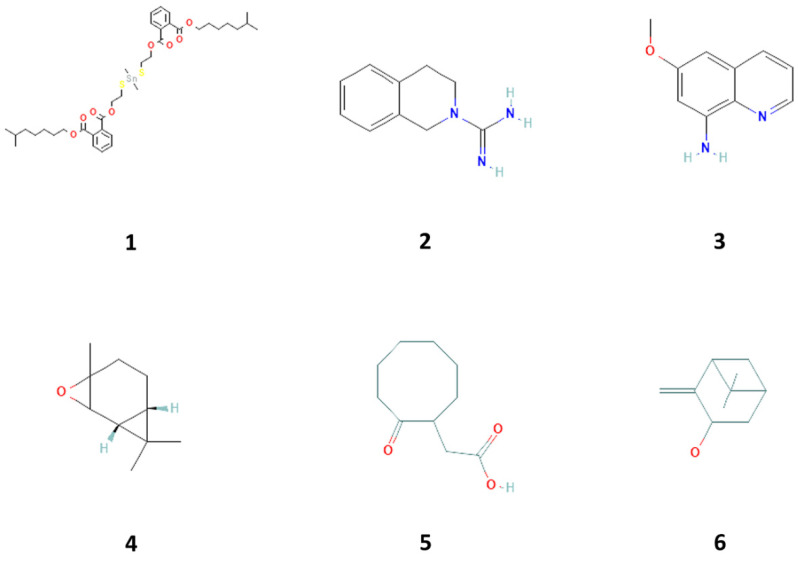
Compounds **1**–**6** isolated from with proliferative biological activity property in sea cucumber (*Holothuria parva*), including 1,2-benzene dicarboxylic acid, diisooctyl ester (**1**), 3,4-dihydro-1h-isoquinoline-2-carboxamidine hydrochloride (**2**), 8-amino-6-methoxyquinoline (**3**), E-2, 3-epoxycarane (**4**), cyclooctaneacetic acid, 2-oxo- (**5**), and isopinocarveol (**6**).

Six compounds showed better affinity to proteins (Table 1, Figure 1). Figures S3–S8 show the 3D plot of the active sites and the 2D plot of the interactions of these proteins with the mentioned compounds. As observed, different interactions, including hydrogen bonds, which are strong van der Waals, and π-π interactions play a role in the binding of compounds to proteins. According to the result of the docking study, the highest affinity to CDK4 was related to 2-cyclohexene-1-ol, 2-methyl-5-(1-methyl phenyl), (Figure S3A). The highest affinity to CDK6 was related to 1,2-benzene dicarboxylic acid, dioctyl ester, and 3,4-dihydro-1h-isoquinoline-2-carboxamidine hydrochloride (Figure S4A,B). The highest affinity to Cyclin D1 was related to 1,2-benzene dicarboxylic acid, dioctyl ester, 3,4-dihydro-1h-isoquinoline-2-carboxamidine hydrochloride, and 8-amino-6-methoxyquinoline (Figure S4). The highest affinity to Cyclin D3 was related to 3,4-dihydro-1h-isoquinoline-2-carboxamidine hydrochloride, isopinocarveol, E-2,3-epoxycarane, 2-cyclohexene-1-ol, 2-methyl-5-(1-methyl phenyl), and Benzene, 2-(butenyl)-5-(1,1-dimethyl ethyl)-1,3-dimethyl- (Figure S5A,B). The highest affinity to cyclin E was related to 1,2-benzene dicarboxylic acid, dioctyl ester, and 3,4-dihydro-1h-isoquinoline-2-carboxamidine hydrochloride (Figure S5C,D). The highest affinity to hypoxia-inducible factor 1 (HIF-1) was related to-6,10-dimethyldodeca-5,9-dien-2-one and 1,2-benzene dicarboxylic acid, dioctyl ester (Figure S6A,B). The highest affinity to TERT (telomerase reverse transcriptase) was related to 1,2-benzene dicarboxylic acid, dioctyl ester, and 3,4-dihydro-1h-isoquinoline-2-carboxamidine hydrochloride. Therefore, six compounds can be considered the most effective ones (Figure S6C,D). The highest affinity to p21 was related to 3,4-dihydro-1h-isoquinoline-2-carboxamidine hydrochloride, azulene and D-glucose, cyclic ethylene mercaptan, pentaacetate, and phenol,2,4-bis(1,1dimethylethyl) (Figure S7A–E). The highest affinity to PCNA was related to-6,10-dimethyldodeca-5,9-dien-2-one, 3,4-dihydro-1h-isoquinoline-2-carboxamidine hydrochloride, and cyclooctaneacetic acid, 2-oxo- (Figure S7A–E).

### 2.4. Morphologic and Immunophenotypic Assays and Differentiation to Adipocytes, Osteocytes, and Chondrocytes Confirmed hUM-MSC Isolation

**Figure 2:**
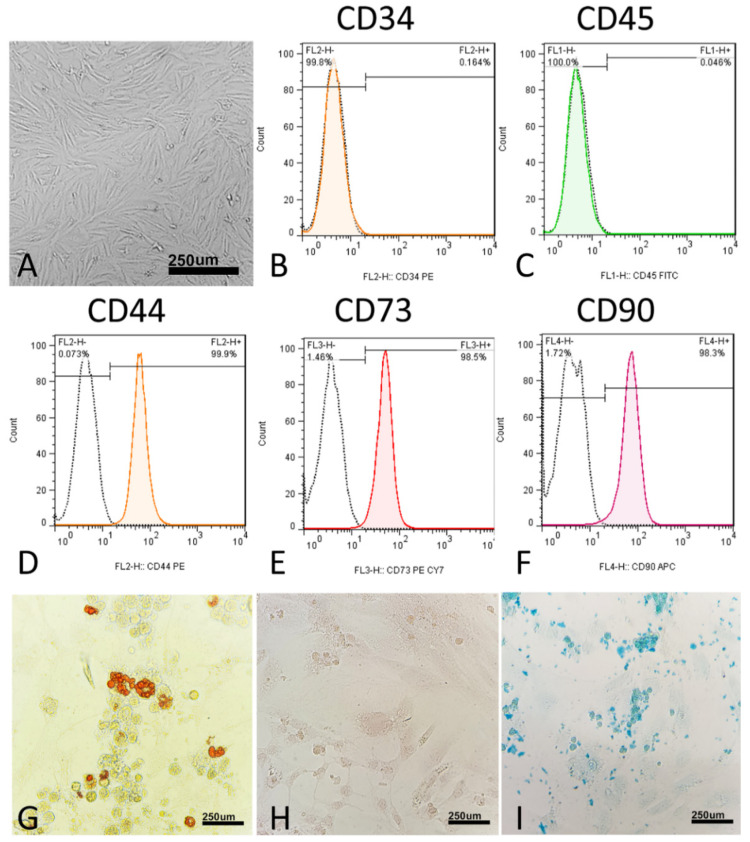
Characterization of human umbilical cord mesenchymal stromal/stem cells (hUC-MSCs). (**A**) hUC-MSCs’ morphology. Flow cytometric histograms showing the immunophenotype of hUC-MSCs. (**B**,**C**) hUC-MSCs are negative for the hematopoietic line markers CD34 and CD45. (**D**–**F**) Analyzed hUC-MSCs are positive for CD44, CD73, and CD90, which are considered to be markers of MSCs. FITC, fluorescein isothiocyanate; PE, phycoerythrin; Cy7, Sulfo-Cyanine7; APC, Allophycocyanin. (**G**–**I**) Differentiation of hUC-MSCs to adipogenic differentiation, in which the cells were stained with oil red O, osteogenic differentiation, in which the cells were stained with alizarin red, and chondrogenic differentiation, in which the cells were stained with Alcian blue.

Morphologically, the MSCs were thin, long, spindle-shaped cells with small cell bodies (Figure 2A). The cells’ morphology did not change during the four passages. At the molecular level, MSCs did not express CD34 (0.04%) and CD45 (0.16%) (Figure 1C and Figure 2B). In addition, at the molecular level, MSCs express surface antigens, including CD44 (99.9%), CD73 (98.5%), and CD90 (98.3%) (Figure 2D–F).

To verify the multipotency of hUC-MSCs, the cells were assessed for their adipogenic, chondrogenic, and osteogenic differentiation capacities in vitro. In the adipogenic differentiated cells, red-stained intracellular vacuoles were observed (Figure 2G). In osteogenic differentiation, adherent monolayers of spindle-shaped cells became multilayered cell clusters surrounded by a matrix-like substance (Figure 2H). In chondrogenic differentiation, on day 21, the cartilaginous elements were numerous and well-differentiated, and the cells were observed under the light microscope for sulfated proteoglycan (Figure 2I).

### 2.5. Aqueous Extract of H. parva Induced Proliferation of hUC-MSCs

**Figure 3:**
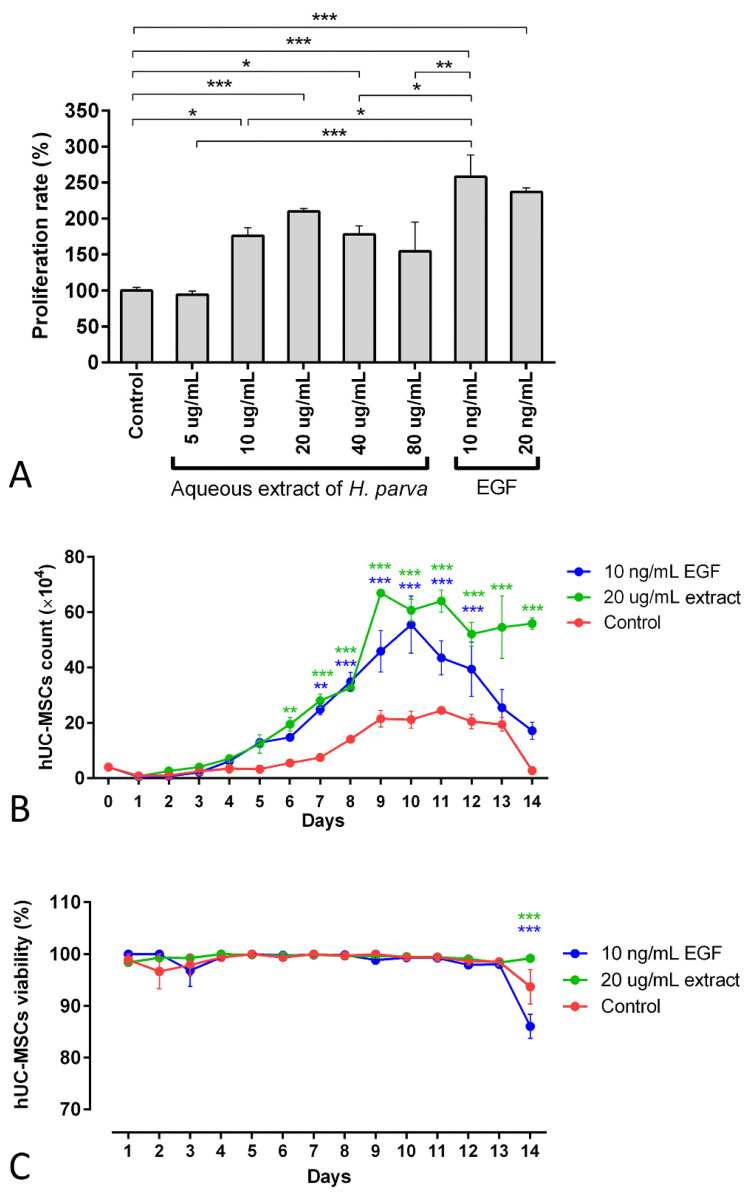
Mean and standard errors of cell proliferation rate MTT (**A**), cell count (**B**), and cell viability (**C**) of human umbilical cord mesenchymal stromal/stem cells (hUC-MSCs) after exposure to the aqueous extract of sea cucumber (*Holothuria parva*) or epidermal growth factor (EGF). * *p* < 0.05; ** *p* < 0.01; *** *p* < 0.001.

The findings of the 3-[4,5-dimethylthiazol-2-yl]-2,5 diphenyl tetrazolium bromide (MTT) assay demonstrated that the 10, 20, and 40 µg/mL aqueous extracts of *H. parva* had proliferative effects on hUC-MSCs (*p* = 0.01, *p* < 0.001, and *p* = 0.014, respectively, Figure 3A). Moreover, EGF-10 and EGF-20, as positive controls, also had proliferative effects on hUC-MSCs (*p* < 0.001, in Figure 3A). In addition, the EGF-10 had higher proliferative effects on hUC-MSCs than the 10 and 40 µg/mL aqueous extract of *H. parva* (*p* < 0.05, Figure 3A). However, the EGF-10 had no statistically significant difference in proliferative effect on hUC-MSCs with the 20 µg/mL of aqueous extract of *H. parva* (Figure 3A). This evidence showed that the 20 µg/mL concentration of the aqueous extract is the optimum dose of sea cucumber extract with minimum cytotoxicity. This concentration was chosen for further analysis.

The number of cells treated with the 20 µg/mL concentration of the extract and EGF-10 increased faster and higher than in the control group (Figure 3B). In detail, the cell number of the extract treatment group was higher than the control group from day 6 (*p* < 0.01). Moreover, this result was observed for the EGF-10 treatment group from day 7 (*p* < 0.01). On the other hand, the 20 µg/mL concentration of aqueous extract of *H. parva* and EGF-10 did not have significant effects on hUC-MSCs’ viability compared to the control group for 13 days (Figure 3C).

### 2.6. Cell Cycle Assay Did Not Show Differences in the Proportion of Stages 

**Figure 4:**
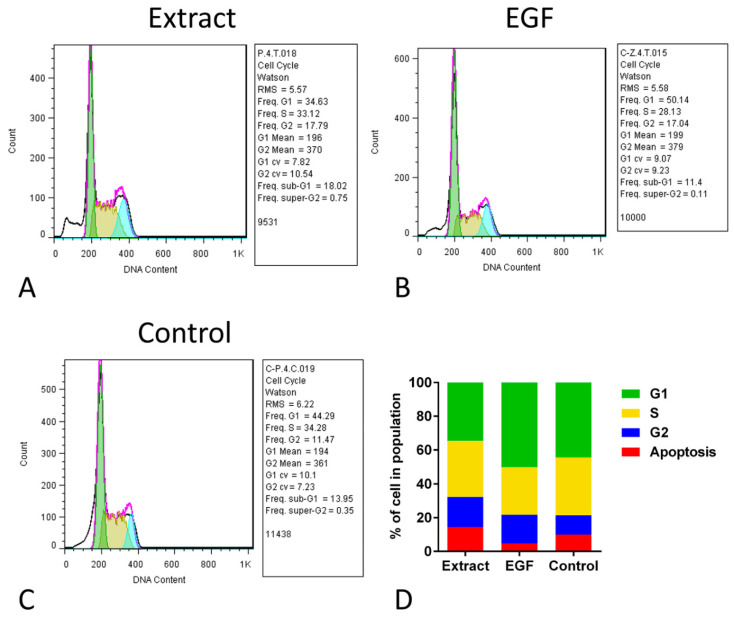
Cell cycle assay of human umbilical cord mesenchymal stromal/stem cells (hUC-MSCs) after exposure to aqueous extract of sea cucumber (*Holothuria parva*) (**A**), epidermal growth factor (EGF) (**B**) or control (**C**). (**D**), Proportion of percent of cell number in four stages of cell cycle in three groups.

The cell cycle assay of hUC-MSCs in the three groups showed that although the percent of cells in the G2 stage of the extract and EGF-10 was mathematically higher than that in the control group, the proportions of different stages in all groups were not statistically different (Figure 4). The frequency of G2 in the extract, EGF, and control groups was 17.79, 17.04, and 11.47, respectively, which were not statistically different. For the frequency of the S stage, the extract, EGF, and control groups were 33.12, 28.13, and 34.28, respectively. In this comparison, the frequency of the S stage in the extract was mathematically higher than the EGF group, indicating more proliferation. In line with previous results, the frequency of the G1 stage in the EGF group was mathematically higher than the extract group (Figure 4).

### 2.7. Western Blot Analysis Findings

**Figure 5:**
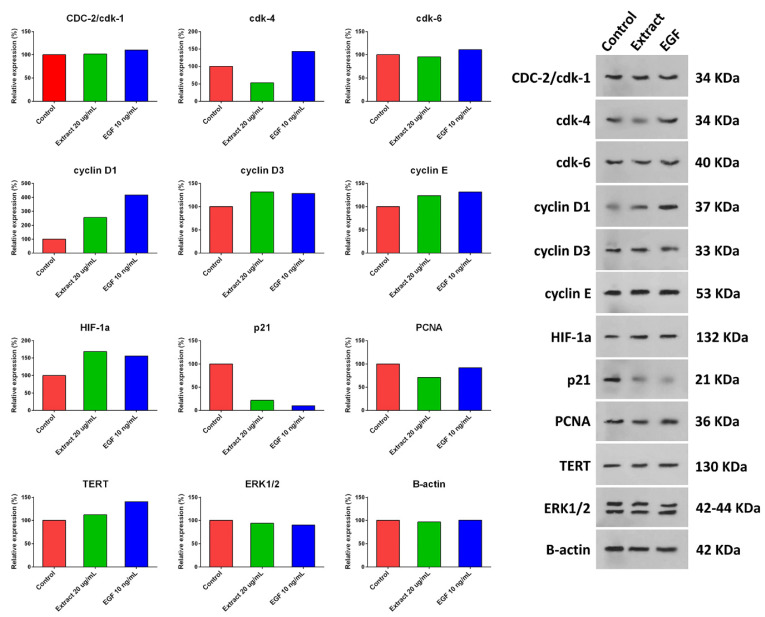
Expression of proliferation-related proteins after treating human umbilical cord mesenchymal stromal/stem cells (hUC-MSCs) with aqueous extract of sea cucumber (*Holothuria parva*) or epidermal growth factor (EGF).

hUC-MSCs were treated with a 20 µg/mL concentration of aqueous extract of *H. parva* or EGF-10, and expression of cyclin D1, cyclin D3, cyclin E, HIF-1α, and TERT were increased compared with the control group (Figure 5 and Figure S9). Moreover, the expression of p21 and PCNA decreased after treating hUC-MSCs with a 20 µg/mL concentration of aqueous extract of *H. parva* or EGF-10 compared with the control group. However, CDC-2/cdk-1 and ERK1/2 had almost the same expression as the control group. The expression of CDK-4 was decreased and increased after treatment with the extract and EGF-10, respectively. In addition, CDK-6 expression was decreased and increased after cell treatment with the extract and EGF-10, respectively.

## 3. Discussion

In the present study, aqueous extract of *H. parva* induced the proliferation of hUC-MSCs, the same as EGF. Growth factors, including EGF, FGF, transforming growth factor beta (TGFβ), and bone morphogenetic protein (BMP), induce proliferation in multipotent MSC cells [35]. These cytokines play roles in the regeneration and development of sea cucumbers [36]. Consistent with our findings, protein fractions from phosphate buffer saline (PBS) and acetic acid crude extracts of *H. scabra* body wall increased the growth kinetics of placenta-derived MSCs [10]. In addition, the pepsin-solubilized collagen extract of *Stichopus japonicus* enhanced human keratinocyte cell proliferation [12]. Water extract of *S. variegatus* induced the proliferation activity of spinal astrocyte cell lines [9]. The optimum concentration of *S. chloronotus* aqueous extract enhanced wound healing in rat models [11].

**Figure 6:**
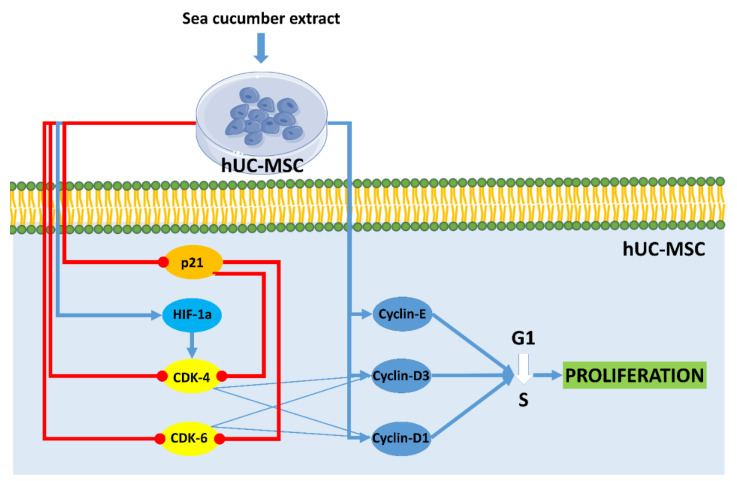
Aqueous extract of sea cucumber (*Holothuria parva*) induces proliferation in human umbilical cord mesenchymal stromal/stem cells (hUC-MSCs). Small colorful dots, red lighting, blue arrows, and red hammer-headlines represent the extract bioactive molecules detected by the docking technique, the extract-induced stimulus, direct reactions, and each inhibiting reaction, respectively.

Cell cycle analysis on hUC-MSCs showed the proportion of cells at the G2 stage after treatment with aqueous extract of *H. parva* and EGF was higher than in the control group. This result indicated that the cells after treatment with both extract and EGF undergo proliferation and they are in the pre-mitosis stage. In line with the current result, *S. horrens* extract induced cell proliferation, increased S and G2 phases, and finally mitosis [37]. Moreover, the effects of aqueous extract of *H. parva* on proliferation-related peptides in hUC-MSCs were assessed. Aqueous extract of *H. parva* or EGF upregulated expression of cyclin D1, cyclin D3, and cyclin E in hUC-MSCs. Cyclin D protein subfamilies cyclin D1 and cyclin D3 play an important role in cell proliferation [38]. They perform this role by activating CDK-4 or CDK-6 [38]. Similar to our findings, EGF induces hair follicle-derived MSC proliferation through the EGFR/ERK and AKT pathways associated with upregulation of cyclin D1 expression and stimulation of the G1/S transition [39]. It is shown that cyclin D3 is expressed in cells stimulated by EGF for a G1 phase progression [40]. Cyclin E forms the CDK2-cyclin E complexes, which both promote G1/S phase progression [41]. On the other hand, aqueous extract of *H. parva* or EGF downregulated the expression of p21 in hUC-MSCs. The p21 protein is a CDK inhibitor and downregulates proliferation by preventing the transcription of cell cycle-regulated pro-proliferative proteins [42]. EGF promotes cell growth by suppressing p21 [43]. Therefore, aqueous extract of *H. parva* is the same as EGF-induced proliferation of hUC-MSCs through upregulation of cyclin subfamilies and suppression of cellular senescence-related proteins (Figure 6). 

Aqueous extract of *H. parva* or EGF upregulated expression of the TERT in hUC-MSCs. TERT maintains telomere length to enable cells to proliferate [44]. TERT mRNA expression increased in hUC-MSCs treated by EGF [45]. TERT expression is high in stem cells during proliferation and reduced upon differentiation [44]. EGF activates TERT transcription in cancer cells but not in somatic cells [46]. Therefore, aqueous extract of *H. parva* is the same as EGF-induced proliferation of hUC-MSCs through maintaining telomere length.

Aqueous extract of *H. parva* or EGF increased the expression of the HIF-1α in hUC-MSCs. HIF-1 has been recognized for its key role in transcriptional control of proliferation [47]. The EGF can stabilize HIF-α under non-hypoxic conditions [48]. Previous studies have pointed out that the EGF could elevate the expression of HIF-1α [49]. Moreover, it was shown that growth factors promote HIF-α binding to DNA to induce gene transcription in cells [50]. Similarly, HIF-1α contributes to the proliferative response of cells to growth factors [51]. Therefore, aqueous extract of *H. parva* is the same as EGF-induced proliferation of hUC-MSCs through maintaining telomere length.

Docking analysis of GC-MS-detected bioactive compounds showed 3,4-dihydro-1h-isoquinoline-2-carboxamidine hydrochloride, which is also named debrisoquine and is an antihypertensive drug [31], has the highest affinity. As observed in Table S2, debrisoquine hydrochloride showed the highest affinity to CDK4, CDK6, cyclin D1, cyclin D3, cyclin E, PCNA, and TERT. The highest affinity of debrisoquine hydrochloride was calculated with cyclin D3. An increase in cyclin D3 was observed after sea cucumber extract exposure. Based on the best of our knowledge, no data show the effects of debrisoquine hydrochloride on cell proliferation. Cyclooctaneacetic acid, 2-oxo- showed the highest affinity to cyclin D3, CDK4, and PCNA. The highest affinity to debrisoquine hydrochloride was calculated with cyclin D3. After sea cucumber extract exposure to MSCs, cyclin D3 increased. Based on the best of our knowledge, no data show the effects of cyclooctaneacetic acid, 2-oxo- on cell proliferation; 8-amino-6-methoxyquinoline has antimalarial and antiplasmodial activity effects [52]; and 8-quinolinamine analogs did not show any cytotoxicity on cancerous and noncancerous cells [53]. However, the hematotoxic effect of a metabolite of 8-amino-6-methoxyquinoline on erythrocytes has been reported [32]; 8-amino-6-methoxyquinoline showed the highest affinity to cyclin D1, cyclin D3, and CDK4. The highest affinity of 8-amino-6-methoxyquinoline was calculated with cyclin D3. There was no data on the effect of this compound on cell growth. Isopinocarveol, which has also been known as pinocarveol, has an anti-viral effect [33,34]. It has been reported that trans-pinocarveol has antigenotoxic potential [54]. Pinocarveol showed the highest affinity to cyclin D3 in our present study. Diisooctyl phthalate, also known as 1,2-benzene dicarboxylic acid, dioctyl ester, has been shown previously in the sea cucumber [55], and 1,2-benzene dicarboxylic acid, dioctyl ester has antioxidant activity [56]. Diisooctyl phthalate had a fungi toxic effect on six fungi and cytotoxic activity on newborn shrimp [57]. Diisooctyl phthalate showed the highest affinity to CDK6, cyclin D1, cyclin E, HIF-1α, PCNA, and TERT. The highest affinity of diisooctyl phthalate was calculated with CDK6. A decrease in CDK6 was observed after sea cucumber extract exposure. E-2, 3-epoxycarane is a terpenoid. E-2 and 3-epoxycarh have anti-aging effects on the skin [58,59]. E-2, 3-epoxycarane showed the highest affinity to cyclin D3 in the present study.

## 4. Materials and Methods

### 4.1. Ethical Approval Statements

This disquisition was performed in agreement with applicable guidelines and regulations for animal studies. All experimental protocols were approved by ethical committee of the Bushehr University of Medical Sciences with permission number: IR.BPUMS.REC.1398.091.

### 4.2. Sampling and Identification of Sea Cucumber

Ten sea cucumbers were used in the current study and weighed 100 gr. They were harvested alive from the coastal waters of Dayyer, the Persian Gulf, Iran (28°57′44.6″ N 50°48′42.5″ E). They were sent to the laboratory in containers filled with fresh seawater for extraction and further processing. They were rinsed with distilled water to remove debris and transferred to the aquarium with a 29 ppt salinity and a 26 °C water temperature. The captured sea cucumbers were identified according to identification keys [22,23]. All the sea cucumbers were identified as *H. parva* species.

### 4.3. Preparation of Sea Cucumber Aqueous Extract

The sea cucumbers identified as *H. parva* were washed with tap water at the laboratory to eliminate all particles from their bodies. The sea cucumber body was cut longitudinally, removing all the internal organs. The body wall was washed with distilled water properly and then cut into small pieces. The samples, which weighed 50 gr, were homogenized using a blender and mixed with distilled water. The prepared mixture was filtered using Whatman 125 mm filter paper. Following filtration, we freeze-dried the liquid for 12 h and stored the final powder, which was about 1 gr at −80 °C [55].

### 4.4. GC-MS Assay

The GC-MS analysis was used to evaluate the chemical composition of the sea cucumber extract [60]. The lyophilized fractions were subjected to the 7890B Agilent Gas Chromatography-Mass Spectroscopy. Electron ionization (EI) mass spectra (scan range, *m/z* 50–500) were obtained using electrons with an energy of 70 eV and filament emission of 0.5 mA. The GC separations were conducted using an HP-5MS UI column (30 m × 0.25 mm i.d., film thickness of 0.5 µm). Helium was used as the carrier gas (flow: 0.8 mL/min) for EI. The GC-MS oven temperature was programmed at 5 °C/min from 80 °C after 3 min since the sample injection and held at 250 °C for 10 min. The injection port of the gas chromatograph, transfer line, and ion source of 5977MSD were maintained at 240 °C, 250 °C, and 220 °C, respectively. The separated compounds were identified by comparing them with the compound data from the National Institute of Standards and Technology (NIST MS database, 2014) library. The relative percent amount of each component was measured by comparing its average peak area to the total areas.

### 4.5. hUC-MSCs Culture and Characterization

hUC-MSCs were commercially purchased (PerciaVista R&D Co. cell bank, Shiraz, Iran). According to the manufacturer’s instructions, hUC-MSCs were isolated from infants’ umbilical cords and cultured [61]. The cells were cultured in 75 cm^2^. Tissue culture flask (NEST, Cat. No. 708003, China) and incubated (Memmert, INB200, Büchenbach, Germany) at 37 °C with 5% CO^2^ in a 95% humidity, cultured in a Dulbecco’s Modified Eagle’s medium (DMEM) (Gibco™, Cat. No. 12-800-082, Cambridge, UK) medium containing 10% FBS (Kiazist, Cat. No. KFBS100, Tehran, Iran), 1% penicillin-streptomycin (Gibco™, Cat. No. 15-140-122, Cambridge, UK) and 1% gentamicin (Sigma-Aldrich, CAS. No1405-41-0, St. Louis, MO, USA) [62].

Flasks of hUC-MSCs were incubated at 4 °C in the dark with either phycoerythrin (PE) or fluorescein isothiocyanate (FITC)-conjugated antibodies specific for CD44, CD90, CD73, CD34, and CD45. Cells were analyzed by flow cytometry using a BD FACS Calibur (BD Biosciences, San Jose, CA, USA) [61].

Adherent cells were subjected to adipogenic, osteogenic, and chondrogenic differentiation in vitro, according to established protocols [63]. In a 24-well cell culture plate, 5 × 10^4^ cells were seeded, and 2 mL of culture medium was added. Then the cells were incubated at 37 °C in a humidified atmosphere of 5% CO_2_. After 24 h, the culture medium was replaced with differentiation media (Kiazist, Tehran, Iran). Cells were treated with differentiation media for three weeks, with medium changes every 3 d. To document the adipogenic differentiation, the culture was rinsed three times with 1X PBS (Sigma-Aldrich, P4417-100TAB, St. Louis, MO, USA) and fixed with 4% formalin (Merck Cat. No. 1040022500, Darmstadt, Germany) for 20 min. Then, the formalin was removed and washed with sterile distilled water and stained for 20 min with oil red O to stain lipid droplets, and alizarin red staining was used to observe the calcium-rich extracellular matrix observed under the microscope (Optika, Cat. No. IM-3FL4, Modena, Italy). For validating chondrogenic differentiation, after removing formalin, the cells were washed with sterile distilled water, and 40 µL of HCl 0.1M (Merck, Darmstadt, Germany) was added. HCl was removed, and 0.5 mL Alcian blue was added. After 20 min, the stain was removed and washed twice with PBS 1X. The cells were observed under a light microscope for glycosaminoglycans.

### 4.6. MTT Proliferation Assay

For the MTT assay, 4 × 10^4^ hUC-MSCs were seeded in each well of a 96-well tissue culture plate (Sorfa, Cat. No. 220400, Zhejiang, China) and 200 µL of culture medium was added, then it incubated for 24 h to let cells attach to the bottom of the plate [64]. After that, replacing the medium with different doses of aqueous extract (5, 10, 20, 40, and 80 µg/mL) after some modification of previous findings [9], EGF (10 and 20 ng/mL) as a positive control [9], and culture media as a negative control. After 72 h of treatment, the medium was removed and washed twice with PBS 1X. Then, 100 μL of MTT solution (bio-idea, BI-2004, Tehran, Iran) was added per well and incubated for 4 h. After that, 50 μL of dimethyl sulfoxide (DMSO, bio-idea, BI-2004, Tehran, Iran) was added. The plate was put in an incubator for 20 min. Quantification was then carried out using a microplate reader at 570 nm.

### 4.7. Cell Count and Cell Viability Assays

For these assays, 1 × 10^4^ hUC-MSCs were seeded in each well of a 24-well cell culture plate, and 2 mL of culture medium was added. Then, it was incubated for 24 h to let cells attach to the bottom of the plate [65]. Then, the medium was replaced with culture media, 20 µg/mL aqueous extract, and 10 ng/mL EGF and replaced every 3 d. These concentrations were selected based on the MTT results. To count the cells, every 24 h, the medium was removed and washed with PBS 1X. Adding 0.5 mL trypsin-EDTA (Gibco™, Cat. No. 25300054, Cambridge, UK) for 4 min of incubation, and then 1.5 mL of culture medium was added for enzyme neutralization. The suspension was centrifuged, and the supernatant was removed. Finally, 1 mL of culture medium was added to the cells and shaken well to create a homogeneous mixture. 10 µL of this mixture was pipetted and mixed with the same volume of trypan blue and counted with a hemocytometer under a light microscope. Based on the point that trypan blue does not penetrate viable cells with intact membranes and appears white, while dead cells were distinguished from viable ones by their blue color. Viability was mentioned by the percentage difference between the control and other groups, same as in the previous study and guideline [66].

### 4.8. Cell Cycle Assay 

Treated hUC-MSCs with 20 µg/mL aqueous extract or 10 ng/mL EGF for 72 h were harvested at 80–90% confluence for cell cycle analysis. The cell concentration was adjusted to 5 × 10^5^ cell/mL. Cells were washed with PBS 1X and were fixed with 70% ethanol at 4 °C for 2 h. Fixed cells were centrifuged, and the supernatant was discarded. The pellet was washed and incubated in a 1 mL:propidium iodide (PI) master mix included 40 μL of PI, 10 μL of RNase (DNase free), and 950 μL of PBS for 30 min. Cell cycles were assessed by flow cytometry, and analysis was performed using FlowJo software (Tree Star Inc., Ashland, Wilmington, DE, USA) [67].

### 4.9. Western Blot

Western blot analysis was done based on the standard procedures with slight modifications [68]. After 72 h of treatment of hUC-MSCs with 20 µg/mL aqueous extract or 10 ng/mL EGF, cells were lysed by lysis buffer, including 500 µL tris-HCL pH = 8, 0.003 gr EDTA, 0.08 gr NaCl, 0.025 gr sodium deoxycholate, and 0.01 gr sodium dodecyl sulfate, one tablet of a protease inhibitor cocktail, and 10 µL NP40 (1%) triton at 4 °C for 20 min. The lysates were centrifuged at 12000× *g* for 10 min at 4 °C, and the protein concentration was measured by a Bradford protein assay. Then, proteins were transferred to a microporous polyvinylidene difluoride membrane (Millipore, Molsheim, France). Membranes were incubated in a blocking buffer for 1 h at room temperature. After blocking, the membranes were incubated with the corresponding primary antibodies separately overnight at 4 °C. 

Immunoblotting was performed with β-actin (c4): sc-47778 (Santa Cruz Biotechnology, San Diego, CA, USA), cdc2 p34 (17): sc-54 (Santa Cruz Biotechnology), CDK-4 (DCS-35): sc-23896 (Santa Cruz Biotechnology), cdk6 (b-10): sc-7961 (Santa Cruz Biotechnology), cyclin D1 (a-12): sc-8396 (Santa Cruz Biotechnology), cyclin D3 (1): sc-135875 (Santa Cruz Biotechnology), cyclin E (he12): sc-247 (Santa Cruz Biotechnology), ERK 1/2 (h-72): sc-292838 (Santa Cruz Biotechnology), HIF-1α (28b): sc-13515 (Santa Cruz Biotechnology), p21 (f-5): sc-6246 (Santa Cruz Biotechnology), PCNA (pc11): sc-53407 (Santa Cruz Biotechnology), and TERT polyclonal antibody e-ab-33070 (Elabscience Biotechnology, Wuhan, China). Membranes were washed 3 times (10 min each time) in tris-buffered saline before incubating with m-IgGκBP-HRP: sc-516102 (Santa Cruz Biotechnology) or mouse anti-rabbit IgG-HRP: sc-2357 (Santa Cruz Biotechnology) secondary antibodies. One of the most accurate and sensitive techniques for detecting the desired protein band (identified by its specific antibody) is the use of chemoluminescence kits. The ECL advanced reagents kit and its protocol were used.

### 4.10. Computational Details 

#### 4.10.1. Preparation of Ligands and Receptors

Fifty-eight compounds were detected through the GC-MS analysis of the aqueous extract of the sea cucumber’s (*H. parva*) body wall. Accordingly, these compounds were selected as ligands based on their interactions with nine target proteins through the docking process. Their three-dimensional (3D) structure was downloaded from the PubChem database. Nine proteins of CDK4, CDK6, cyclin D1, cyclin D3, cyclin E, HIF-1α, p21, PCNA, and TERT were downloaded from the Protein Database Bank (PDB) with PDB codes of 2w96, 1blx, 2w96, 3g33, 7kjs, 4h6j, 5e0u, 5e0u, and 5ugw. The HyperChem software version 8.0.10 was used to optimize the geometry of the ligands. All receptors were prepared for the docking process using Chimera 1.15 (University of California, San Francisco, CA, USA). 

#### 4.10.2. Generation of a Grid Box

The grid box was manually generated with a space of 0.375 Å at the position of the active sites chosen according to the result of the CASTp calculation.

#### 4.10.3. Study of Target Proteins Marine-Derived Compound Interactions

To perform the docking process, Autodock Vina 1.1.2 software (Scripps Research, San Diego, CA, USA) was utilized to investigate interactions between receptors and ligands.

### 4.11. Statistical Analysis

The data were statistically analyzed using IBM SPSS Statistics 26 software (SPSS for Windows, version 26, SPSS Inc., Chicago, IL, USA). Comparison between groups was done using one-way ANOVA and the post hoc LSD test (for comparing the MTT and cell proliferation and viability assays) or the chi-square test (for comparing the cell cycle analysis and flow cytometry). Data were demonstrated as mean ± standard error. The significant difference between groups was statistically considered *p* < 0.05. The graphs were drawn using GraphPad Prism (v7.0a, GraphPad Software, Inc., San Diego, CA, USA).

## 5. Conclusions

In line with the current study, previous investigations of sea cucumber suggested that its bioactive compounds could be potentially useful in the drug discovery area. It could be one of the first steps in further exploration in medical approaches of marine compounds [21]. In the current study, it was indicated that the aqueous extracts of sea cucumber, *H. parva*, same as EGF, may be able to induce proliferation in hUC-MSCs through different mechanisms, such as upregulation of cyclin subfamilies, suppression of cellular senescence-related protein, and maintenance of the telomere length. In details, the MTT, cell viability, and PDT assays showed proliferative effects of sea cucumber aqueous extract. However, in comparison with EGF, no better results were achieved totally, but in some analyses, better results of the extract were shown. Moreover, the GC-MS detected six effective bioactive compounds in sea cucumber aqueous extract that had the property of inducing stem cell proliferation, as demonstrated by computational analysis. 

## Figures and Tables

**Table 1:** Gas chromatography-mass spectrometry (GC-MS)-detected and docking computational analysis confirmed compounds with proliferative biological activity properties in sea cucumber (*Holothuria parva*) based on the PubChem database.

No.	Compounds	Formula	MW (g/mol)	Peak Height	Retention Time (min)	Peak Area	References
1	1,2-benzene dicarboxylic acid, diisooctyl ester	C24H38O4	390.6	36645	39.618	1.538	[30]
2	3,4-dihydro-1h-isoquinoline-2-carboxamidine hydrochloride	C10H13N3	175.23	258766	6.726	4.358	[31]
3	8-amino-6-methoxyquinoline	C10H10N2O	174.2	27202	14.114	1.063	[32]
4	E-2, 3-epoxycarane	C10H16O	152.23	86504	6.479	1.756	ND
5	Cyclooctaneacetic acid, 2-oxo-	C10H16O3	184.23	5310	28.246	0.317	ND
6	Isopinocarveol	C10H16O	152.23	57697	5.964	0.453	[33,34]

ND, no data.

## Data Availability

Data are contained within the article or Supplementary materials.

## Supplementary Materials

The following are available online at https://www.mdpi.com/article/10.3390/md21050267/s1, Figure S1: The image of ossicles isolated from sea cucumber (*Holothuria parva*), Figure S2: GC-MS analysis of aqueous extract of sea cucumber (*Holothuria parva*), Figure S3: (A) The 3D plot of the binding sites and the 2D plot of interactions of CDK4 with Benzene 1-ethyl- 3-methyl-, the 3D plot of the binding sites, and the 2D plot of interactions of CDK6 with (B) 1, 2-benzene dicarboxylic acid, diisooctyl ester, and (C) 3,4-Dihydro-1h-isoquinoline-2-carboxamidine hydrochloride, Figure S4: The 3D plot of the binding sites and the 2D plot of interactions of Cyclin D1 with (A) 1, 2-benzene dicarboxylic acid, diisooctyl ester, (B) 3,4-Dihydro-1h-isoquinoline-2-carboxamidine hydrochloride, (C) 8-amino-6-methoxyquinoline, and (D) Cycloundecene,1-methyl, Figure S5: The 3D plot of the binding sites and the 2D plot of interactions of Cyclin D3 with (A) 3,4-Dihydro-1h-isoquinoline-2-carboxamidine hydrochloride, (B) Cycloundecene,1-methyl-. The 3D plot of the binding sites and the 2D plot of interactions of Cyclin E with (C) 1, 2-benzene dicarboxylic acid, diisooctyl ester, and (D) 3,4-Dihydro-1h-isoquinoline-2-carboxamidine hydrochloride, Figure S6: The 3D plot of the binding sites and the 2D plot of interactions of HIF-1α with (A) 6,10-Dimethyldodeca-5,9-dien-2-one, and (B) 1, 2-benzene dicarboxylic acid, diisooctyl ester. The 3D plot of the binding sites and the 2D plot of interactions of TERT with (C) 1, 2-benzene dicarboxylic acid, diisooctyl ester, and (D) 3,4-Dihydro-1h-isoquinoline-2-carboxamidine hydrochloride, Figure S7: The 3D plot of the binding sites and the 2D plot of interactions of p21 with (A) 3,4-Dihydro-1h-isoquinoline-2-carboxamidine hydrochloride, (B) Azulene, (C) Benzene 1-ethyl-3-methyl-, (D) D-glucose, cyclic ethylene mercaptan, pentaacetate, and E) Phenol,2,4-bis(1,1dimethylethyl)-, Figure S8: The 3D plot of the binding sites and the 2D plot of interactions of PCNA with (A) (5E,9E)-6,10-Dimethyldodeca-5,9-dien-2-one, (B) 3,4-Dihydro-1h-isoquinoline-2-carboxamidine hydrochloride, (C) Benzene 1-ethyl-3-methyl-, (D) Cyclooctaneacetic acid, 2-oxo-, and (E) Cycloundecene,1-methyl-, Figure S9: The original gels of western blot analysis, Table S1: GC-MS compounds in aqueous extract of sea cucumber (*Holothuria parva*) based on PubChem database, Table S2: The values of binding affinity (Kcal/mol) of ligands to receptors [24,25,26,27,28,29,30,31,32,33,34,69,70,71,72,73,74,75,76,77,78,79,80,81,82,83,84,85,86,87,88,89,90,91,92,93,94,95,96,97,98,99,100,101,102,103,104,105,106,107,108].

## References

[1] Xie Q., Liu R., Jiang J., Peng J., Yang C., Zhang W., Wang S., Song J. (2020). What is the impact of human umbilical cord mesenchymal stem cell transplantation on clinical treatment?. Stem Cell Res. Ther..

[2] Jo H., Brito S., Kwak B.M., Park S., Lee M.G., Bin B.H. (2021). Applications of mesenchymal stem cells in skin regeneration and rejuvenation. Int. J. Mol. Sci..

[3] Rice P., Orgill D. (2019). Assessment and Classification of Burn Injury. Uptodate.

[4] Tottoli E.M., Dorati R., Genta I., Chiesa E., Pisani S., Conti B. (2020). Skin wound healing process and new emerging technologies for skin wound care and regeneration. Pharmaceutics.

[5] Ornelas-González A., Chairez-Cantu K., Ortiz-Martínez M., González-González M., Rito-Palomares M. (2021). Stem cell culture media enriched with plant-derived compounds: Cell proliferation enhancement. J. Chem. Technol. Biotechnol..

[6] Ormond D.R., Shannon C., Oppenheim J., Zeman R., Das K., Murali R., Jhanwar-Uniyal M. (2014). Stem cell therapy and curcumin synergistically enhance recovery from spinal cord injury. PLoS ONE.

[7] San Miguel-Ruiz J.E., Garcia-Arraras J.E. (2007). Common cellular events occur during wound healing and organ regeneration in the sea cucumber *Holothuria glaberrima*. BMC Dev. Biol..

[8] Khotimchenko Y. (2018). Pharmacological potential of sea cucumbers. Int. J. Mol. Sci..

[9] Patar A., Jamalullail S., Jaafar H., Abdullah J.M. (2012). The effect of water extract of sea cucumber *Stichopus variegatus* on rat spinal astrocytes cell lines. Curr. Neurobiol..

[10] Kornthong N., Saengsuwan J., Duangprom S., Songkoomkrong S., Vivattanasarn T., Suwansa-ard S., Manochantr S., Sobhon P. (2020). The effects of sea cucumber extract (*Holothuria scabra*) on human mesenchymal stem cells derived from placenta. J. Med. Assoc. Thai..

[11] Mazliadiyana M., Nazrun A., Isa N. (2017). Optimum dose of sea cucumber (*Stichopus chloronotus*) extract for wound healing. Med. Health.

[12] Park S.Y., Lim H.K., Lee S., Hwang H.C., Cho S.K., Cho M. (2012). Pepsin-solubilised collagen (PSC) from Red Sea cucumber (*Stichopus japonicus*) regulates cell cycle and the fibronectin synthesis in HaCaT cell migration. Food Chem..

[13] Li Q., Cai C., Chang Y., Zhang F., Linhardt R.J., Xue C., Li G., Yu G. (2018). A novel structural fucosylated chondroitin sulfate from *Holothuria mexicana* and its effects on growth factors binding and anticoagulation. Carbohydr. Polym..

[14] Zhang Y., Song S., Song D., Liang H., Wang W., Ji A. (2010). Proliferative effects on neural stem/progenitor cells of a sulfated polysaccharide purified from the sea cucumber *Stichopus japonicus*. J. Biosci. Bioeng..

[15] Sheng X., Li M., Song S., Zhang N., Wang Y., Liang H., Wang W., Ji A. (2012). Sulfated polysaccharide isolated from the sea cucumber Stichopus japonicus promotes neurosphere migration and differentiation via up-regulation of N-cadherin. Cell. Mol. Neurobiol..

[16] Arundina I., Suardita K., Setiabudi H., Ariani M.D. (2016). Golden sea cucumbers (*Stichopus Hermanii*) as growth factors of stem cells. J. Int. Dent. Med. Res..

[17] Kazuhiro H., Suriadi O., Nakagami G., Oe M., Nakatani T., Okuwa M., Sanada H., Sugama J. (2017). A prospective observational study using sea cucumber and honey as topical therapy for diabetic foot ulcers in Indonesia. J. Wellness Health Care.

[18] Wang Y., Su W., Zhang C., Xue C., Chang Y., Wu X., Tang Q., Wang J. (2012). Protective effect of sea cucumber (*Acaudina molpadioides*) fucoidan against ethanol-induced gastric damage. Food Chem..

[19] Fahmy S.R., Amer M.A., Al-killidar M.H. (2015). Ameliorative effect of the sea cucumber *Holothuria arenicola* extract against gastric ulcer in rats. J. Basic Appl. Zool..

[20] Eisapour M., Salari Aliabadi M.A., Salamat N., Nafisi Bahabadi M., Salati A.P. (2022). Identification and taxonomy of sea cucumbers (*Holothuria*) in Persian Gulf. Iran. J. Fish. Sci..

[21] Pangestuti R., Arifin Z. (2018). Medicinal and health benefit effects of functional sea cucumbers. J. Tradit. Complement. Med..

[22] Purcell S., Samyn Y., Conand C. (2012). Commercially Important Sea Cucumbers of the World.

[23] Samyn Y., Vandenspiegel D., Massin C. (2006). Taxonomie des Holothuries des Comores.

[24] Terekhova E.A., Stepicheva N.A., Pshenichnikova A.B., Shvets V.I. (2010). Stearic acid methyl ether: A new extracellular metabolite of the obligate methylotrophic bacterium Methylophilus quaylei. Prikl. Biokhim. Mikrobiol..

[25] Othman A.R., Abdullah N., Ahmad S., Ismail I.S., Zakaria M.P. (2015). Elucidation of in-vitro anti-inflammatory bioactive compounds isolated from *Jatropha curcas* L. plant root. BMC Complement. Altern. Med..

[26] Chen P.Y., Wu C.Y., Clemons G.A., Citadin C.T., Couto E.S.A., Possoit H.E., Azizbayeva R., Forren N.E., Liu C.H., Rao K.N.S. (2020). Stearic acid methyl ester affords neuroprotection and improves functional outcomes after cardiac arrest. Prostaglandins Leukot. Essent. Fat. Acids.

[27] Panagiotopoulos A., Tseliou M., Karakasiliotis I., Kotzampasi D.M., Daskalakis V., Kesesidis N., Notas G., Lionis C., Kampa M., Pirintsos S. (2021). p-cymene impairs SARS-CoV-2 and influenza A (H1N1) viral replication: In silico predicted interaction with SARS-CoV-2 nucleocapsid protein and H1N1 nucleoprotein. Pharmacol. Res. Perspect..

[28] Formiga R.O., Alves Junior E.B., Vasconcelos R.C., Araujo A.A., de Carvalho T.G., de Araujo Junior R.F., Guerra G.B.C., Vieira G.C., de Oliveira K.M., Diniz M. (2021). Effect of p-cymene and rosmarinic acid on gastric ulcer healing—Involvement of multiple endogenous curative mechanisms. Phytomedicine Int. J. Phytother. Phytopharm..

[29] Hassan S.M.H., Ray P., Hossain R., Islam M.T., Salehi B., Martins N., Sharifi-Rad J., Amarowicz R. (2020). p-Cymene metallo-derivatives: An overview on anticancer activity. Cell Mol. Biol..

[30] Chen S., Liu J., Gong H., Yang D. (2009). Identification and antibacterial activity of secondary metabolites from Taxus endophytic fungus. Chin. J. Biotechnol..

[31] Idle J.R., Mahgoub A., Angelo M.M., Dring L.G., Lancaster R., Smith R.L. (1979). The metabolism of [14C]-debrisoquine in man. Br. J. Clin. Pharmacol..

[32] Bolchoz L.J., Budinsky R.A., McMillan D.C., Jollow D.J. (2001). Primaquine-induced hemolytic anemia: Formation and hemotoxicity of the arylhydroxylamine metabolite 6-methoxy-8-hydroxylaminoquinoline. J. Pharmacol. Exp. Ther..

[33] Yadalam P.K., Varatharajan K., Rajapandian K., Chopra P., Arumuganainar D., Nagarathnam T., Sohn H., Madhavan T. (2021). Antiviral essential oil components against SARS-CoV-2 in pre-procedural mouth rinses for dental settings during COVID-19: A computational study. Front. Chem..

[34] El Yaagoubi M., Ortiz S., Mechqoq H., Cavaleiro C., Lecso-Bornet M., Rodrigues M.J., Custodio L., El Mousadik A., Grougnet R., El Aouad N. (2021). Chemical composition, antibacterial screening and cytotoxic activity of *Chiliadenus antiatlanticus* (Emb. & Maire) Gómiz (Asteraceae) essential oil. Chem. Biodivers..

[35] Rodrigues M., Griffith L.G., Wells A. (2010). Growth factor regulation of proliferation and survival of multipotential stromal cells. Stem Cell Res. Ther..

[36] Sun L., Chen M., Yang H., Wang T., Liu B., Shu C., Gardiner D.M. (2011). Large scale gene expression profiling during intestine and body wall regeneration in the sea cucumber *Apostichopus japonicus*. Comp. Biochem. Physiol. Part D Genom. Proteom..

[37] Pilus N.S., Muhamad A., Shahidan M.A., Yusof N.Y. (2022). Potential of epidermal growth factor-like peptide from the sea cucumber *Stichopus horrens* to increase the growth of human cells: In silico molecular docking approach. Mar. Drugs.

[38] Pestell R.G. (2013). New roles of cyclin D1. Am. J. Pathol..

[39] Bai T., Liu F., Zou F., Zhao G., Jiang Y., Liu L., Shi J., Hao D., Zhang Q., Zheng T. (2017). Epidermal growth factor induces proliferation of hair follicle-derived mesenchymal stem cells through epidermal growth factor receptor-mediated activation of ERK and AKT signaling pathways associated with upregulation of cyclin D1 and downregulation of p16. Stem Cells Dev..

[40] Depoortere F., Van Keymeulen A., Lukas J., Costagliola S., Bartkova J., Dumont J.E., Bartek J., Roger P.P., Dremier S. (1998). A requirement for cyclin D3-cyclin-dependent kinase (cdk)-4 assembly in the cyclic adenosine monophosphate-dependent proliferation of thyrocytes. J. Cell Biol..

[41] Shen H., Zhou E., Wei X., Fu Z., Niu C., Li Y., Pan B., Mathew A.V., Wang X., Pennathur S. (2015). High density lipoprotein promotes proliferation of adipose-derived stem cells via S1P1 receptor and Akt, ERK1/2 signal pathways. Stem Cell Res. Ther..

[42] Arthur L.M., Heber-Katz E. (2011). The role of p21 in regulating mammalian regeneration. Stem Cell Res. Ther..

[43] Alexander P.B., Yuan L., Yang P., Sun T., Chen R., Xiang H., Chen J., Wu H., Radiloff D.R., Wang X.-F. (2015). EGF promotes mammalian cell growth by suppressing cellular senescence. Cell Res..

[44] Mattson M.P., Zhang P., Fu W. (2013). Roles for TERT and telomerase in cell differentiation and apoptosis. Madame Curie Bioscience Database [Internet].

[45] Salehinejad P., Alitheen N.B., Mandegary A., Nematollahi-Mahani S.N., Janzamin E. (2013). Effect of EGF and FGF on the expansion properties of human umbilical cord mesenchymal cells. Vitr. Cell Dev. Biol. Anim..

[46] Bermudez Y., Yang H., Cheng J.Q., Kruk P.A. (2008). Pyk2/ERK 1/2 mediate Sp1- and c-Myc-dependent induction of telomerase activity by epidermal growth factor. Growth Factors.

[47] Tekin D., Dursun A.D., Xi L. (2010). Hypoxia inducible factor 1 (HIF-1) and cardioprotection. Acta Pharmacol. Sin..

[48] Sun J., Shen H., Shao L., Teng X., Chen Y., Liu X., Yang Z., Shen Z. (2020). HIF-1alpha overexpression in mesenchymal stem cell-derived exosomes mediates cardioprotection in myocardial infarction by enhanced angiogenesis. Stem Cell Res. Ther..

[49] Jeong W., Bazer F.W., Song G., Kim J. (2016). Expression of hypoxia-inducible factor-1 by trophectoderm cells in response to hypoxia and epidermal growth factor. Biochem. Biophys. Res. Commun..

[50] Richard D.E., Berra E., Pouyssegur J. (2000). Nonhypoxic pathway mediates the induction of hypoxia-inducible factor 1alpha in vascular smooth muscle cells. J. Biol. Chem..

[51] Schultz K., Fanburg B.L., Beasley D. (2006). Hypoxia and hypoxia-inducible factor-1alpha promote growth factor-induced proliferation of human vascular smooth muscle cells. Am. J. Physiol. Heart Circ. Physiol..

[52] Hochegger P., Dolensky J., Seebacher W., Saf R., Kaiser M., Maser P., Weis R. (2021). 8-amino-6-methoxyquinoline-tetrazole hybrids: Impact of linkers on antiplasmodial activity. Molecules.

[53] Jain M., Reddy C.R.P., Halder M., Singh S., Kumar R., Wasudeo S.G., Singh P.P., Khan S.I., Jacob M.R., Tekwani B.L. (2018). Synthesis and biological evaluation of 8-quinolinamines and their amino acid conjugates as broad-spectrum anti-infectives. ACS Omega.

[54] Drosopoulou E., Vlastos D., Efthimiou I., Kyrizaki P., Tsamadou S., Anagnostopoulou M., Kofidou D., Gavriilidis M., Mademtzoglou D., Mavragani-Tsipidou P. (2018). In vitro and in vivo evaluation of the genotoxic and antigenotoxic potential of the major Chios mastic water constituents. Sci. Rep..

[55] Keshavarz M., Shamsizadeh F., Tavakoli A., Baghban N., Khoradmehr A., Kameli A., Rasekh P., Daneshi A., Nabipour I., Vahdat K. (2021). Chemical compositions and experimental and computational modeling activity of sea cucumber *Holothuria parva* ethanolic extract against herpes simplex virus type 1. Biomed. Pharmacother..

[56] Li M., Zhou L., Yang D., Li T., Li W. (2012). Biochemical composition and antioxidant capacity of extracts from *Podophyllum hexandrum* rhizome. BMC Complement. Altern. Med..

[57] Shafiqur Rahman M.D., Anwar M.N. (2006). Fungitoxic and cytotoxic activity of a novel compound 1,2-benzenedicarboxylic acid, diisooctyl ester of *Plumbago zeylanica* Linn. Asian J. Microbiol. Biotechnol. Environ. Sci..

[58] Vertuani S., Beghelli E., Scalambra E., Malisardi G., Copetti S., Dal Toso R., Baldisserotto A., Manfredini S. (2011). Activity and stability studies of verbascoside, a novel antioxidant, in dermo-cosmetic and pharmaceutical topical formulations. Molecules.

[59] Young E., Godwin J. (2019). Assessing olive, palm kernel, and groundnut oils for their dermatologically-active agents. Int. J. Sci. Res. Sci. Technol..

[60] Mohebbi G., Nabipour I., Vazirizadeh A., Vatanpour H., Farrokhnia M., Maryamabadi A., Bargahi A. (2018). Acetylcholinesterase inhibitory activity of a neurosteroidal alkaloid from the upside-down jellyfish *Cassiopea andromeda* venom. Rev. Bras. Farm..

[61] Rezaeian L., Hosseini S.E., Dianatpour M., Edalatmanesh M.A., Tanideh N., Mogheiseh A., Tamadon A. (2018). Intrauterine xenotransplantation of human Wharton jelly-derived mesenchymal stem cells into the liver of rabbit fetuses: A preliminary study for in vivo expression of the human liver genes. Iran J. Basic Med. Sci..

[62] Kadam S., Govindasamy V., Bhonde R. (2012). Generation of Functional Islets from Human Umbilical Cord and Placenta Derived Mesenchymal Stem Cells. Somatic Stem Cells.

[63] Bazoobandi S., Tanideh N., Rahmanifar F., Zare S., Koohi-Hosseinabadi O., Razeghian-Jahromi I., Dianatpour M., Ahmadi M., Khoradmehr A., Nabipour I. (2020). Preventive effects of intrauterine injection of bone marrow-derived mesenchymal stromal cell-conditioned media on uterine fibrosis immediately after endometrial curettage in rabbit. Stem Cells Int..

[64] Zeng H.L., Zhong Q., Qin Y.L., Bu Q.Q., Han X.A., Jia H.T., Liu H.W. (2011). Hypoxia-mimetic agents inhibit proliferation and alter the morphology of human umbilical cord-derived mesenchymal stem cells. BMC Cell Biol..

[65] Tamadon A., Mehrabani D., Zarezadeh Y., Rahmanifar F., Dianatpour M., Zare S. (2017). Caprine endometrial mesenchymal stromal stem cell: Multilineage potential, characterization, and growth kinetics in breeding and anestrous stages. Vet. Med. Int..

[66] Kamiloglu S., Sari G., Ozdal T., Capanoglu E. (2020). Guidelines for cell viability assays. Food Front..

[67] Zhang H., Li Z.L., Yang F., Zhang Q., Su X.Z., Li J., Zhang N., Liu C.H., Mao N., Zhu H. (2018). Radial shockwave treatment promotes human mesenchymal stem cell self-renewal and enhances cartilage healing. Stem Cell Res. Ther..

[68] Yip W.K., Cheenpracha S., Chang L.C., Ho C.C., Seow H.F. (2010). Anti-proliferative and anti-invasive properties of a purified fraction from *Streptomyces* sp. H7372. Int. J. Oncol..

[69] Hakozaki T., Laughlin T., Zhao S., Wang J., Deng D., Jewell-Motz E., Elstun L. (2013). A regulator of ubiquitin-proteasome activity, 2-hexyldecanol, suppresses melanin synthesis and the appearance of facial hyperpigmented spots. Br. J. Dermatol..

[70] Shirani M., Samimi A., Kalantari H., Madani M., Kord Zanganeh A. (2017). Chemical composition and antifungal effect of hydroalcoholic extract of *Allium tripedale* (Tvautv.) against *Candida* species. Curr. Med. Mycol..

[71] Figueiredo C.R., Matsuo A.L., Massaoka M.H., Girola N., Azevedo R.A., Rabaca A.N., Farias C.F., Pereira F.V., Matias N.S., Silva L.P. (2014). Antitumor activity of kielmeyera coriacea leaf constituents in experimental melanoma, tested in vitro and in vivo in syngeneic mice. Adv. Pharm. Bull..

[72] Vambe M., Naidoo D., Aremu A.O., Finnie J.F., Van Staden J. (2021). Bioassay-guided purification, GC-MS characterization and quantification of phyto-components in an antibacterial extract of *Searsia lancea* leaves. Nat. Prod. Res..

[73] Lim C.S., Wong W.F., Rosli R., Ng K.P., Seow H.F., Chong P.P. (2009). 2-dodecanol (decyl methyl carbinol) inhibits hyphal formation and SIR2 expression in *C. albicans*. J. Basic Microbiol..

[74] Rajendran B.K., Xavier Suresh M., Bhaskaran S.P., Harshitha Y., Gaur U., Kwok H.F. (2018). Pharmacoinformatic approach to explore the antidote potential of phytochemicals on bungarotoxin from Indian krait, *Bungarus caeruleus*. Comput. Struct. Biotechnol. J..

[75] Witkowska-Banaszczak E., Długaszewska J. (2017). Essential oils and hydrophilic extracts from the leaves and flowers of *Succisa pratensis* Moench. and their biological activity. J. Pharm. Pharmacol..

[76] Doukas P.H., Speaker T.J., Thompson R.S. (1975). Azulene analogs of pharmacological agents III: Acute toxicity and local anesthetic activity of azulylamides and azulenecarboxamides. J. Pharm. Sci..

[77] Chen C.H., Lee O., Yao C.N., Chuang M.Y., Chang Y.L., Chang M.H., Wen Y.F., Yang W.H., Ko C.H., Chou N.T. (2010). Novel azulene-based derivatives as potent multi-receptor tyrosine kinase inhibitors. Bioorg. Med. Chem. Lett..

[78] Aoki S., Ohta K., Matsumoto K., Sakai H., Abe M., Miura M., Sugawara F., Sakaguchi K. (2006). An emulsion of sulfoquinovosylacylglycerol with long-chain alkanes increases its permeability to tumor cells. J. Membr. Biol..

[79] Dhouibi R., Moalla D., Ksouda K., Ben Salem M., Hammami S., Sahnoun Z., Zeghal K.M., Affes H. (2018). Screening of analgesic activity of Tunisian *Urtica dioica* and analysis of its major bioactive compounds by GCMS. Arch. Physiol. Biochem..

[80] Thabet A.A., Youssef F.S., El-Shazly M., AN B.S. (2020). GC-MS and GC-FID analyses of the volatile constituents of *Brachychiton rupestris* and Brachychiton discolor, their biological activities and their differentiation using multivariate data analysis. Nat. Prod. Res..

[81] Herman S., Kny A., Schorn C., Pfatschbacher J., Niederreiter B., Herrmann M., Holmdahl R., Steiner G., Hoffmann M.H. (2012). Cell death and cytokine production induced by autoimmunogenic hydrocarbon oils. Autoimmunity.

[82] George J.D., Price C.J., Marr M.C., Myers C.B., Schwetz B.A., Heindel J.J. (1998). Evaluation of the developmental toxicity of methacrylamide and N,N’-methylenebisacrylamide in Swiss mice. Toxicol. Sci..

[83] Mozaffarian D., Cao H., King I.B., Lemaitre R.N., Song X., Siscovick D.S., Hotamisligil G.S. (2010). Circulating palmitoleic acid and risk of metabolic abnormalities and new-onset diabetes. Am. J. Clin. Nutr..

[84] Zhang L., Lv J., Chen C., Wang X. (2021). Roles of acyl-CoA synthetase long-chain family member 5 and colony stimulating factor 2 in inhibition of palmitic or stearic acids in lung cancer cell proliferation and metabolism. Cell Biol. Toxicol..

[85] Bi C., Zhang T., Li Y., Zhao H., Zhang P., Wang Y., Xu Y., Gu K., Liu Y., Yu J. (2020). A proteomics- and metabolomics-based study revealed that disorder of palmitic acid metabolism by aconitine induces cardiac injury. Chem. Res. Toxicol..

[86] Sanchez-Alegria K., Bastian-Eugenio C.E., Vaca L., Arias C. (2021). Palmitic acid induces insulin resistance by a mechanism associated with energy metabolism and calcium entry in neuronal cells. FASEB J..

[87] Galindo-Hernandez O., Leija-Montoya A.G., Romero-Garcia T., Vazquez-Jimenez J.G. (2021). Palmitic acid decreases cell migration by increasing RGS2 expression and decreasing SERCA expression. Genet. Mol. Biol..

[88] Korbecki J., Bajdak-Rusinek K. (2019). The effect of palmitic acid on inflammatory response in macrophages: An overview of molecular mechanisms. Inflamm. Res..

[89] Marrez D.A., Naguib M.M., Sultan Y.Y., Higazy A.M. (2019). Antimicrobial and anticancer activities of *Scenedesmus obliquus* metabolites. Heliyon.

[90] Rajkumar S., Jebanesan A. (2004). Mosquitocidal activities of octacosane from *Moschosma polystachyum* Linn (lamiaceae). J. Ethnopharmacol..

[91] Jing L.L., He L., Fan P.C., Jia Z.P., Ma H.P. (2015). Chemical constituents with anti-hypoxia activity from *Saussurea involucrata*. J. Chin. Med. Mat..

[92] Figueiredo C.R., Matsuo A.L., Pereira F.V., Rabaca A.N., Farias C.F., Girola N., Massaoka M.H., Azevedo R.A., Scutti J.A., Arruda D.C. (2014). *Pyrostegia venusta* heptane extract containing saturated aliphatic hydrocarbons induces apoptosis on B16F10-Nex2 melanoma cells and displays antitumor activity in vivo. Pharmacogn. Mag..

[93] Tátrai E., Ungváry G., Cseh I.R., Mányai S., Szeberényi S., Molnár J., Morvai V. (1981). The effect of long-term inhalation of ortho-xylene on the liver. Industrial and Environmental Xenobiotics.

[94] Sarma S.N., Kim Y.J., Song M., Ryu J.C. (2011). Induction of apoptosis in human leukemia cells through the production of reactive oxygen species and activation of HMOX1 and Noxa by benzene, toluene, and o-xylene. Toxicology.

[95] Varsha K.K., Devendra L., Shilpa G., Priya S., Pandey A., Nampoothiri K.M. (2015). 2,4-di-tert-butyl phenol as the antifungal, antioxidant bioactive purified from a newly isolated *Lactococcus* sp.. Int. J. Food Microbiol..

[96] Zhao F., Wang P., Lucardi R.D., Su Z., Li S. (2020). Natural sources and bioactivities of 2,4-Di-Tert-butylphenol and its analogs. Toxins.

[97] Chathuranga K., Weerawardhana A., Dodantenna N., Ranathunga L., Cho W.K., Ma J.Y., Lee J.S. (2021). Inhibitory effect of *Sargassum fusiforme* and its components on replication of respiratory syncytial virus in vitro and in vivo. Viruses.

[98] Paudel M.R., Chand M.B., Pant B., Pant B. (2019). Assessment of antioxidant and cytotoxic activities of extracts of *Dendrobium crepidatum*. Biomolecules.

[99] Uddin S.J., Grice D., Tiralongo E. (2012). Evaluation of cytotoxic activity of patriscabratine, tetracosane and various flavonoids isolated from the Bangladeshi medicinal plant *Acrostichum aureum*. Pharm. Biol..

[100] Muhammad F., Monteiro-Riviere N.A., Riviere J.E. (2005). Comparative in vivo toxicity of topical JP-8 jet fuel and its individual hydrocarbon components: Identification of tridecane and tetradecane as key constituents responsible for dermal irritation. Toxicol. Pathol..

[101] Sharma R., Locke B.R. (2010). Jet fuel toxicity: Skin damage measured by 900-MHz MRI skin microscopy and visualization by 3D MR image processing. Magn. Reson. Imaging.

[102] Zazula R., Moravec M., Pehal F., Nejtek T., Protus M., Muller M. (2021). Myristic Acid Serum Levels and Their Significance for Diagnosis of Systemic Inflammatory Response, Sepsis, and Bacteraemia. J. Pers. Med..

[103] Khalil A.S.M., Giribabu N., Yelumalai S., Shahzad H., Kilari E.K., Salleh N. (2021). Myristic acid defends against testicular oxidative stress, inflammation, apoptosis: Restoration of spermatogenesis, steroidogenesis in diabetic rats. Life Sci..

[104] Kim Y.G., Lee J.H., Park S., Kim S., Lee J. (2022). Inhibition of polymicrobial biofilm formation by saw palmetto oil, lauric acid and myristic acid. Microb. Biotechnol..

[105] Singh S., Singh J. (2003). Percutaneous absorption, biophysical, and macroscopic barrier properties of porcine skin exposed to major components of JP-8 jet fuel. Environ. Toxicol. Pharmacol..

[106] Singh S., Singh J. (2004). Dermal toxicity and microscopic alterations by JP-8 jet fuel components in vivo in rabbit. Environ. Toxicol. Pharmacol..

[107] Choi D., Kang W., Park T. (2020). Anti-allergic and anti-inflammatory effects of undecane on mast cells and keratinocytes. Molecules.

[108] Nagella P., Ahmad A., Kim S.J., Chung I.M. (2012). Chemical composition, antioxidant activity and larvicidal effects of essential oil from leaves of *Apium graveolens*. Immunopharmacol. Immunotoxicol..

