# Supplementary material for: Proliferative Effect of Aqueous Extract of Sea Cucumber (Holothuria parva) Body Wall on Human Umbilical Cord Mesenchymal Stromal/Stem Cells
Source: Mar Drugs. 2023 Apr 26;21(5):267. doi: 10.3390/md21050267 (PMC10220604; doi:10.3390/md21050267)
Supplement: Supplementary file 1 [file marinedrugs-21-00267-s001.zip › marinedrugs-2061987-supplementary.pdf]

Proliferative effect of aqueous extract of sea cucumber (*Holothuria parva*) body wall on human umbilical cord mesenchymal stromal/stem cells

Poorya Rasekh[1,*], Ali Kameli[1,*], Arezoo Khoradmehr[1,*], Neda Baghban[1,*], Gholamhossein Mohebbi[1], Alireza Barmak[2], Iraj Nabipour[1], Hossein Azari[1], Yaser Heidari[1], Adel Daneshi[1], Afshar Bargahi[1], Zahra Khodabandeh[3], Shahrokh Zare[3], Alireza Afshar[1], Reza Shirazi[4], Sahar Almasi-Turk[5†], Amin Tamadon[6†]

1- The Persian Gulf Marine Biotechnology Research Center, The Persian Gulf Biomedical Sciences Research Institute, Bushehr University of Medical Sciences, Bushehr, Iran
2- Food Lab, Bushehr University of Medical Sciences, Bushehr, Iran
3- Stem Cells Technology Research Center, Shiraz University of Medical Sciences, Shiraz, Iran
4- Department of Anatomy, School of Medical Sciences, Medicine, UNSW Sydney, Sydney, Australia
5- Department of Anatomical Sciences, Bushehr University of Medical Sciences, Bushehr, Iran
6- PerciaVista R&D Co. Shiraz, Iran

* These authors have same contributions as the first author.

[†] Corresponding authors:
Amin Tamadon, DVM, PhD; PerciaVista R&D Co. Shiraz, Iran; Postal Code: 7514633196; Tel/fax: +98-77-3332-8724; Email: amintamaddon@yahoo.com
Sahar Almasi-Turk, PhD; Department of Anatomical Sciences, Bushehr University of Medical Sciences, Bushehr, Iran; Postal Code: 7518759577; Tel./fax: +98-77-3332-0657 Email: s.almasi@bpums.ac.ir

Supplementary Tables
Table S1.
GC-MS compounds in aqueous extract of sea cucumber (*Holothuria parva*) based on PubChem database

Compounds	Formula	Effects	MW (g/mol)	Peak height	Retention time (min)	Peak area (% of total)	References
1,2-benzene dicarboxylic acid, diisooctyl ester	C24H38O4	Anti-microbial	390.6	36645	39.618	1.538	[1]
10-methyl-e-11-tridecen-1-ol propionate	C17H32O2	ND	268.4	51849	4.839	0.791	ND
1-decanol, 2-hexyl-	C16H34O	Proteolytic activity	242.44	367705	5.334	6.740	[2]
1-decanol, 2-methyl-	C11H24O	ND	172.31	19121	4.902	0.179	ND
1-decen-4-yne, 2-nitro-	C10H15NO2	ND	181.23	42656	6.076	0.902	ND
1-eicosanol	C20H42O	Anti-microbial<br>Anti-cancer	298.5	54353	6.209	0.965	[3,4]
1-heptadecanol	C17H36O	ND	256.5	33725	5.571	0.666	ND
1-nonadecanol	C19H40O	Anti-microbial	284.5	5795	19.059	0.626	[5]
1-octanol 2-butyl-	C12H26O	ND	186.33	56288	5.099	0.986	ND
3,4-Dihydro-1h-isoquinoline-2-carboxamidine hydrochloride	C10H13N3	Anti-hypertensive	175.23	258766	6.726	4.358	[6]
2-(prop-2-enoyloxy)tetradecane	C17H32O2	ND	268.4	35233	5.720	0.346	ND
2-azido-2,4,4,6,6-pentamethylheptane	C12H25N3	ND	211.35	52670	5.744	0.916	ND
2-cyclohexane-1-ol,2-methyl-5-(1-methyl phenyl)	C10H16O	ND	152.23	55737	6.766	1.448	ND
2-dodecanol	C12H26O	Anti-microbial<br>Anti-dotes for bungarotoxin	186.33	9041	8.652	0.244	[7,8]
2-hexyl-1-octanol	C14H30O	Anti-microbial<br>Anti-oxidant	214.39	4685	29.206	0.386	[9]

3-hexadecyloxycarbonyl-5-(2-hydroxyethyl)-4-methylimidazolium ion	C24H45N2O3+	ND	409.6	18208	4.770	0.258	ND
5, 9-dodecadien-2-one, 6, 10-dimethyl-, (e, e))-	C14H24O	ND	208.34	241082	4.562	4.164	ND
8-amino-6-methoxyquinoline	C10H10N2O	Hemotoxic	174.2	27202	14.114	1.063	[10]
Octadecanoic acid, methyl ester	C19H38O2	Growth stimulator<br>Anti-inflammatory<br>Inhibit the detrimental effects of cerebral ischemia	298.5	57390	28.025	3.665	[11-13]
Azulene	C10H8	Cytotoxic<br>Tyrosine kinase inhibitors	128.17	53555	6.906	0.807	[14,15]
Benzene, 1-methyl-4-(1-methylethenyl)-	C10H14	Anti-ulcer<br>Gastric healing activities<br>Anti-viral<br>Anti-cancer	134.22	125471	5.990	1.827	[16-18]
Benzene, 2-(butenyl)-5-(1, 1-dimethyl ethyl)-1, 3-dimethyl-	C16H24	ND	216.36	15636	17.768	0.665	ND
Cyclooctaneacetic acid, 2-oxo-	C10H16O3	ND	184.23	5310	28.246	0.317	ND
Decane	C10H22	Anti-cancer	142.28	58264	5.500	0.759	[19]
2,3,5,8-tetramethyldecane	C14H30	ND	198.39	42243	5.753	0.477	ND
Decane 4-methyl-	C11H24	ND	156.31	62319	5.065	0.622	ND
D-glucose, cyclic ethylene mercaptan, pentaacetate	C18H26O10S2	ND	466.5	33044	5.618	0.703	ND
Dodecanal	C12H24O	ND	184.32	38990	8.931	1.218	ND
2,6,11-trimethyldodecane	C15H32	Analgesic activity	212.41	149747	6.242	2.915	[20]
E-2, 3-epoxycarane	C10H16O	ND	152.23	86504	6.479	1.756	ND
Eicosane	C20H42	ND	282.5	28943	18.886	1.347	ND
Heptacosane	C27H56	Cytotoxic<br>Anti-microbial	380.7	35417	40.291	1.502	[9,21]
5-ethyl-2,2,3-trimethyl heptane	C12H26	ND	170.33	42335	5.248	0.690	ND

Hexadecane	C16H34	Cytotoxic	226.44	6471	13.946	0.468	[22]
Isopinocarveol	C10H16O	Anti-viral<br>Anti-microbial	152.23	57697	5.964	0.453	[23,24]
Methoxyacetic acid, 2-tetradecyl ester	C17H34O3	ND	286.4	52858	5.789	0.913	ND
Methoxyacetic acid, 3-tridecyl ester	C16H32O3	ND	272.42	6340	39.815	0.230	ND
Methoxyacetic acid, pentadecyl ester	C18H36O3	ND	300.5	38470	5.048	0.563	ND
Methyl 3-hydroxy-tetra decanoate	C15H30O3	ND	258.4	59301	4.213	0.807	ND
N, n'-methylenebisacrylamide	C7H10N2O2	Adverse maternal and developmental effects	154.17	74866	12.983	2.945	[25]
N-hexadecanoic acid	C16H32O2	Insulin resistance<br>Anti-proliferative<br>Inhibitory effect on cells<br>Inhibited activation of the NLR signaling pathway<br>Reduces cell migration<br>Inflammatory responses	256.42	38564	23.928	2.604	[26-31]
Nonadecane	C19H40	Anti-microbial<br>Anti-cancer	268.5	15779	8.371	0.484	[32]
Octacosane	C28H58	Mosquitocidal activities<br>Prolong the survival time of hypoxic mice<br>Cytotoxic	394.8	67463	38.925	2.421	[33-35]
O-xylene	C8H10	Poisoning (liver disease)<br>Apoptosis induction	106.16	19366	4.654	0.305	[36,37]
Pentadecanoic acid, 14-methyl-, methyl ester	C17H34O2	ND	270.5	35889	22.804	1.732	ND
Pentanoic acid, octyl ester	C13H26O2	ND	214.34	206221	6.026	3.144	ND

Phenol,2,4-bis(1,1dimethylethyl)-	C14H22O	Anti-fungal<br>Cytotoxic<br>Anti-oxidant<br>Anti-microbial<br>Anti-viral	206.32	145200	10.847	5.325	[38,39]
Sulfurous acid, 2-ethylhexyl nonyl ester	C17H36O3S	ND	320.5	3329	16.038	0.160	ND
Tetracosane	C24H50	Anti-viral<br>Cytotoxic	338.7	74354	37.266	2.948	[40-42]
Tetradecane	C14H30	Erythema<br>Cytotoxic	198.39	207453	8.759	5.007	[43,44]
Tetradecanoic acid	C14H28O2	Marker of severe inflammation and sepsis<br>Bacteraemia marker<br>Anti-apoptosis<br>Anti-biofilm activity	228.37	17717	8.171	0.572	[45-47]
Tridecane	C13H28	Systemic toxicity<br>Transepidermal water loss	184.36	183679	7.137	4.238	[48,49]
Undecane	C11H24	Anti-allergic<br>Anti-inflammatory<br>Inhibited degranulation and the secretion of histamine and tumor necrosis factor α (TNF-α)	156.31	166794	7.091	3.100	[50]
Z-10-tetradecen-1-ol acetate	C16H30O2	Anti-oxidant	254.41	57150	5.283	1.114	[51]

ND, no data

Table S2.
The values of binding affinity (Kcal/mol) of ligands to receptors

Compounds	CDK4	CDK6	Cyclin D1	Cyclin D3	Cyclin E	HIF-1α	p21	PCNA	TERT
(5E,9E)-6,10-Dimethyldodeca-5,9-dien-2-one	-6.3	-5.1	-5.4	-4.4	-5.9	-5.8	-3.6	-6	-5.4
1, 2-benzene dicarboxylic acid, diisooctyl ester	-5.2	-7	-6.3	-5.1	-6.6	-5.7	-3.3	-5.4	-6.3
10-methyl-e-11-tridecen-1-ol propionate	-5.2	-4.7	-5.2	-4.1	-4.7	-4.5	-2.9	-4.9	-4.3
1-decanol, 2-hexyl-	-5.2	-4.8	-5.2	-4.4	-4.7	-4.4	-2.9	-5.3	-4.7
1-decanol, 2-methyl-	-5	-4.7	-5	-4.1	-4.4	-3.9	-2.7	-4.8	-4.6
1-decen-4-yne, 2-nitro-	-5.6	-5	-4.9	-4.9	-4.9	-4.3	-3.5	-5.7	-5.2
1-eicosanol	-5.1	-3.9	-4.4	-3.8	-4.2	-3.8	-2.7	-4.6	-4.5
1-heptadecanol	-4.8	-4.4	-4.7	-4	-4.5	-4.2	-3	-4.4	-4.7
1-nonadecanol	-3.9	-4.3	-4.4	-4.1	-4.2	-3.6	-2.7	-5	-4.2
2-(prop-2- enoyloxy)tetradecane	-5.2	-5.5	-5.3	-4.5	-5.2	-3.7	-3.1	-5.6	-4.8
2,3,5,8-tetramethyldecane	-5.1	-4.4	-5.2	-4.2	-4.9	-4	-3	-4.8	-4.7
2,6,11-trimethyldodecane	-5.5	-4.4	-4.9	-4.2	-4.3	-4.2	-3.2	-4.7	-4.4
2-azido-2,4,4,6,6-pentamethylheptane	-5.2	-5.4	-5.4	-5.6	-4.8	-4.1	-3.6	-5.4	-4.9
2-butyl-1-octanol	-5.2	-5	-5.1	-5.6	-4.7	-4	-2.8	-4.7	-3.8
2-Cyclohexen-1-ol, 2-methyl-5-(1-methylethenyl)-, (1S)-	-6	-5.5	-5.6	-6.6	-5.3	-4.1	-3.7	-5.5	-5.2

2-dodecanol	-4.9	-4.6	-4.6	-4.3	-4.6	-3.8	-2.9	-5.1	-4.7
2-hexyl-1-octanol	-5.2	-4.7	-4.9	-6.1	-4.3	-4.7	-2.9	-4.8	-4.6
3,4-Dihydro-1h-isoquinoline-2-carboxamidine hydrochloride	-6.4	-6.6	-6.6	-7.4	-6.3	-3.8	-4.4	-6.4	-6.3
3-hexadecyloxycarbonyl-5-(2-hydroxyethyl)-4-methylimidazolium ion	-4.6	-4.8	-4.5	-4.8	-5.1	-4.3	-2.8	-5.3	-4.5
5-ethyl-2,2,3-trimethylheptane	-5.2	-5.2	-4.9	-4.5	-4.8	-4.7	-3.3	-5.1	-4.3
8-amino-6-methoxyquinoline	-6.3	-5.7	-6.5	-7.1	-5.8	-4	-3.6	-5.5	-5.4
Azulene	-5.8	-5.6	-5.9	-6.8	-5.8	-4.9	-4.2	-5.6	-5.3
Benzene, 1-methyl-4-(1-methylethenyl)-	-5.9	-5.5	-5.4	-5.4	-5	-4.7	-3.8	-5.3	-5.2
Benzene, 2-(butenyl)-5-(1, 1-dimethylethyl)-1, 3-dimethyl-	-5.4	-5.2	-5.2	-6.1	-5.2	-4.5	-3.7	-5.3	-5.3
Cyclooctaneacetic acid, 2-oxo-	-6.4	-5.7	-5.9	-7.3	-5.9	-4.6	-3.7	-6.2	-5.2
Decane	-4.8	-4.4	-4.4	-4.2	-4.4	-3.1	-2.6	-5.1	-4.4
Decane 4-methyl-	-4.6	-4	-4	-3.9	-4.5	-3.6	-2.5	-4.8	-4.4
D-glucose, cyclic ethylene mercaptan, pentaacetate	-5.9	-6.3	-5.6	-4.3	-5.7	-4.2	-4.1	-5.3	-4.8
Dodecanal	-4.7	-4.6	-4.3	-3.8	-4.1	-3.8	-3	-4.9	-4.4
E-2, 3-epoxycarane	-5.9	-5.7	-5.7	-7	-5.5	-4.3	-3.2	-5.5	-4.9
Eicosane	-5.1	-4.5	-4.4	-3.7	-4.5	-3.7	-2.9	-4.4	-4.5

Heptacosane	-3.8	-4.3	-4.7	-3.9	-4.3	-3.5	-2.6	-4.1	-4.5
Hexadecane	-4.7	-4.3	-4.6	-4.5	-4.1	-3.6	-3	-4.6	-4.5
Isopinocarveol	-5.5	-5.4	-5.3	-7.1	-5.5	-4.3	-3.3	-5.2	-5
Methoxyacetic acid, 2-tetradecyl ester	-5.3	-4.8	-4.9	-4.3	-4.8	-4.1	-3.2	-4.8	-4.6
Methoxyacetic acid, 3-tridecyl ester	-5.2	-4.6	-4.7	-3.9	-4.8	-4.1	-3	-4.8	-4.6
Methoxyacetic acid, pentadecyl ester	-4.4	-4.2	-4.7	-3.7	-4.7	-3.9	-2.9	-4.8	-4.2
Methyl 3-hydroxytetradecanoate	-5.4	-4.6	-4.8	-4.4	-4.6	-4.4	-2.8	-4.8	-4.5
N,n'-methylenebisacrylamide	-5.1	-4.4	-4.6	-4.6	-4.7	-3.6	-3.1	-4.5	-4.2
N-hexadecanoic acid	-4.5	-4.6	-4.8	-3.9	-4.5	-3.8	-3.2	-4.8	-4.9
Nonadecane	-4.6	-4.1	-4.2	-4	-4.1	-3.6	-2.6	-4.5	-4.9
Octacosane	-4.5	-3.8	-4.5	-3.9	-4	-3.8	-2.8	-4.6	-4.8
Octadecanoic acid, methyl ester	-5.5	-4.3	-4.6	-5.8	-4.4	-3.8	-2.9	-4.8	-5
O-xylene	-5	-5	-5	-4.1	-5	-4.2	-3.6	-4.7	-4.5
Pentadecanoic acid, 14-methyl-, methyl ester	-5.2	-4.1	-4.6	-4	-4.6	-3.9	-2.9	-5	-4.8
Pentanoic acid, octyl ester	-4.9	-4.3	-4.5	-5.2	-4.7	-3.6	-2.9	-4.6	-4.5
Phenol,2,4-bis(1,1dimethylethyl)-	-6.3	-5.8	-6	-5.9	-5.6	-5	-4.1	-6.3	-5.5
Sulfurous acid, 2-ethylhexyl nonyl ester	-4.6	-4.7	-5	-3.8	-4.6	-4.3	-2.6	-4.6	-4.6

Tetracosane	-3.5	-4.9	-4.4	-3.9	-4.5	-3.7	-2.7	-4.9	-5.1
Tetradecane	-4.7	-4.1	-4.3	-4.2	-4.5	-3.6	-2.8	-4.5	-4.5
Tetradecanoic acid	-5.2	-4.4	-4.8	-5.5	-4.3	-3.7	-3.3	-5.1	-4.4
Tridecane	-4.7	-4.1	-4.1	-3.9	-4.2	-3.4	-2.5	-4.9	-4.4
Undecane	-4.8	-4.3	-4.2	-4.1	-4.3	-3.6	-2.6	-5	-4.1
Z-10-tetradecen-1-ol acetate	-5.3	-4.5	-4.8	-4.4	-4.5	-3.9	-3.2	-5	-5

Supplementary Figures

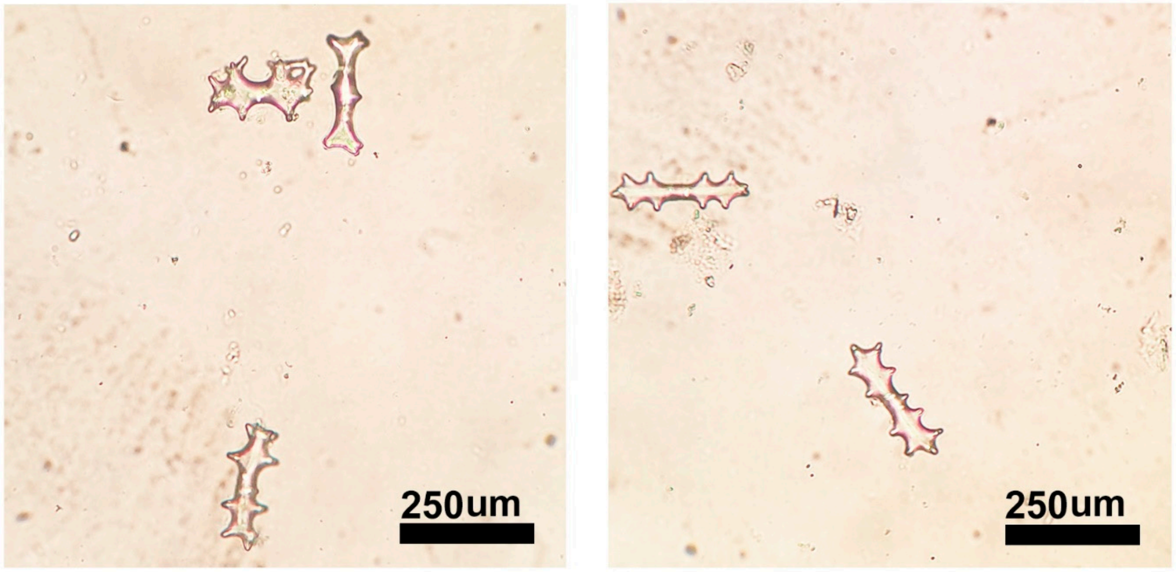

Figure S1.
The image of ossicles isolated from sea cucumber (*Holothuria parva*)

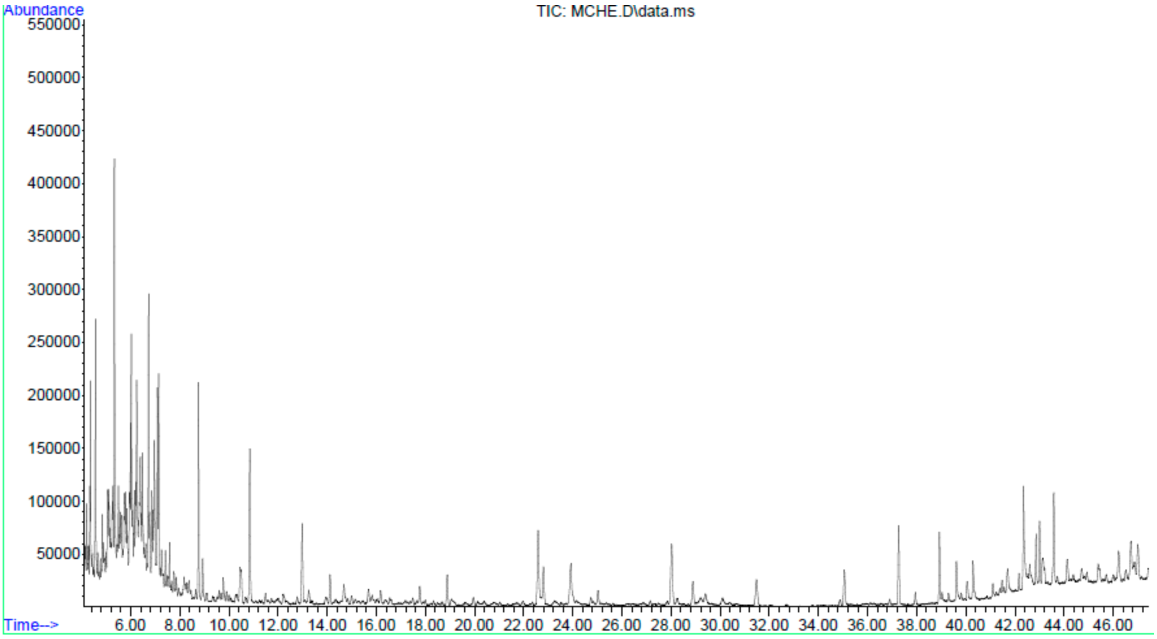

Figure S2.
GC-MS analysis of aqueous extract of sea cucumber (*Holothuria parva*).

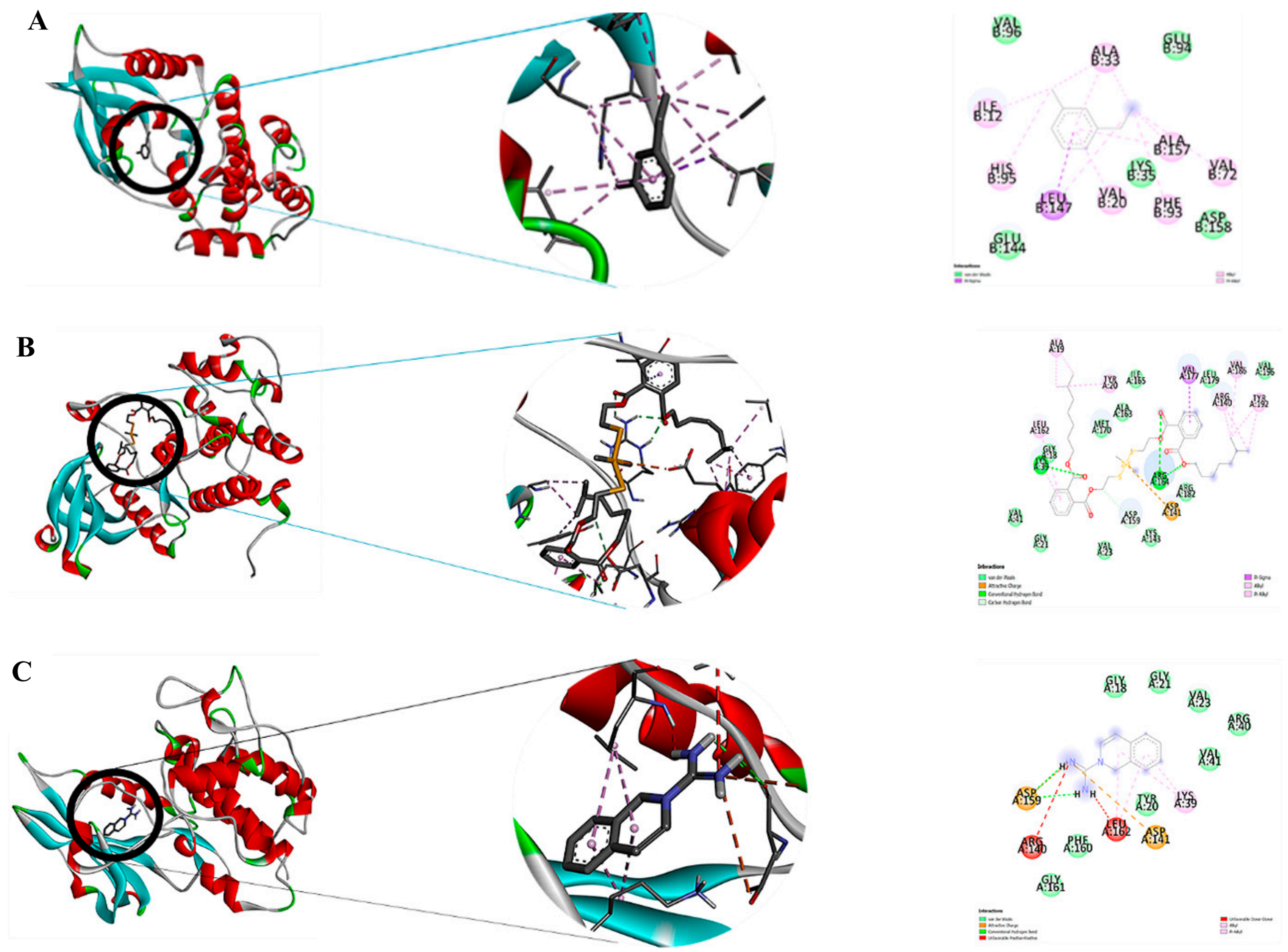

Figure S3.
A) The 3D plot of the binding sites and the 2D plot of interactions of CDK4 with Benzene 1-ethyl- 3-methyl-, the 3D plot of the binding sites, and the 2D plot of interactions of CDK6 with B) 1, 2-benzene dicarboxylic acid, diisooctyl ester, and C) 3,4-Dihydro-1h-isoquinoline-2-carboxamidine hydrochloride.

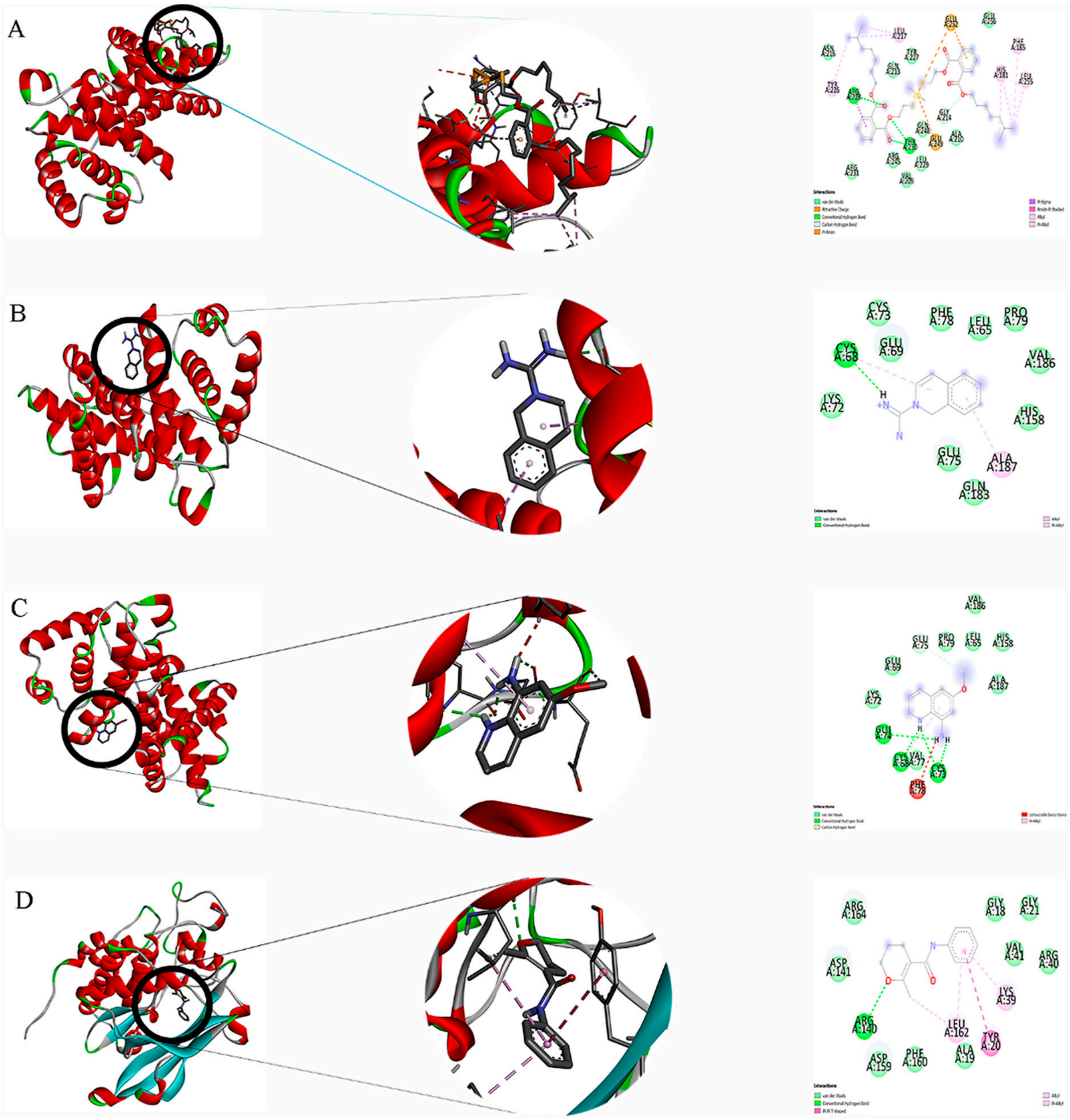

Figure S4.
The 3D plot of the binding sites and the 2D plot of interactions of Cyclin D1 with A) 1, 2-benzene dicarboxylic acid, diisooctyl ester, B) 3,4-Dihydro-1h-isoquinoline-2-carboxamidine hydrochloride, C) 8-amino-6-methoxyquinoline, and D) Cycloundecene,1-methyl.

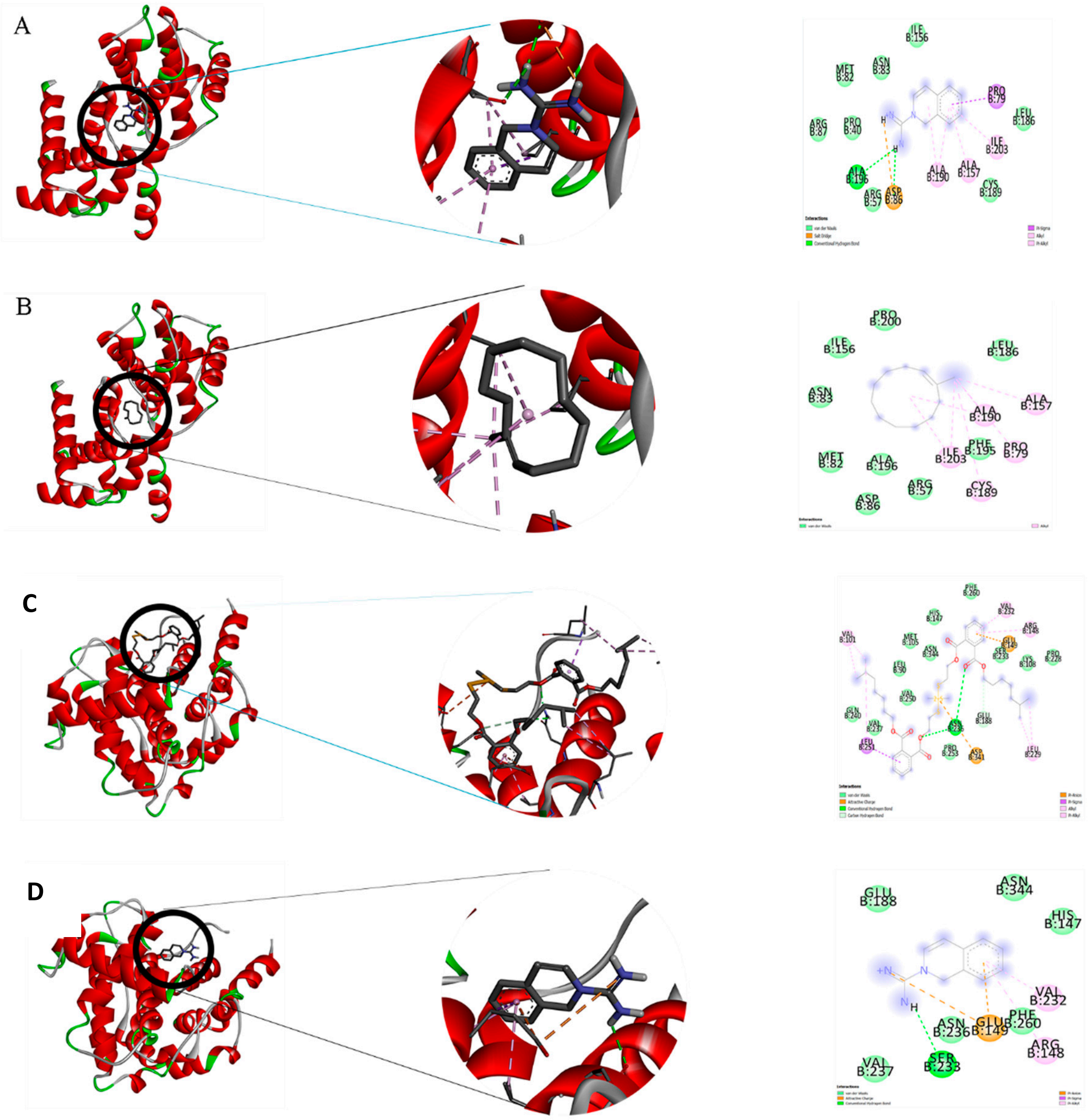

Figure S5.

The 3D plot of the binding sites and the 2D plot of interactions of Cyclin D3 with A) 3,4-Dihydro-1h-isoquinoline-2-carboxamidine hydrochloride, B) Cycloundecene,1-methyl-. The 3D plot of the binding sites and the 2D plot of interactions of Cyclin E with C) 1, 2-benzene dicarboxylic acid, diisooctyl ester, and D) 3,4-Dihydro-1h-isoquinoline-2-carboxamidine hydrochloride.

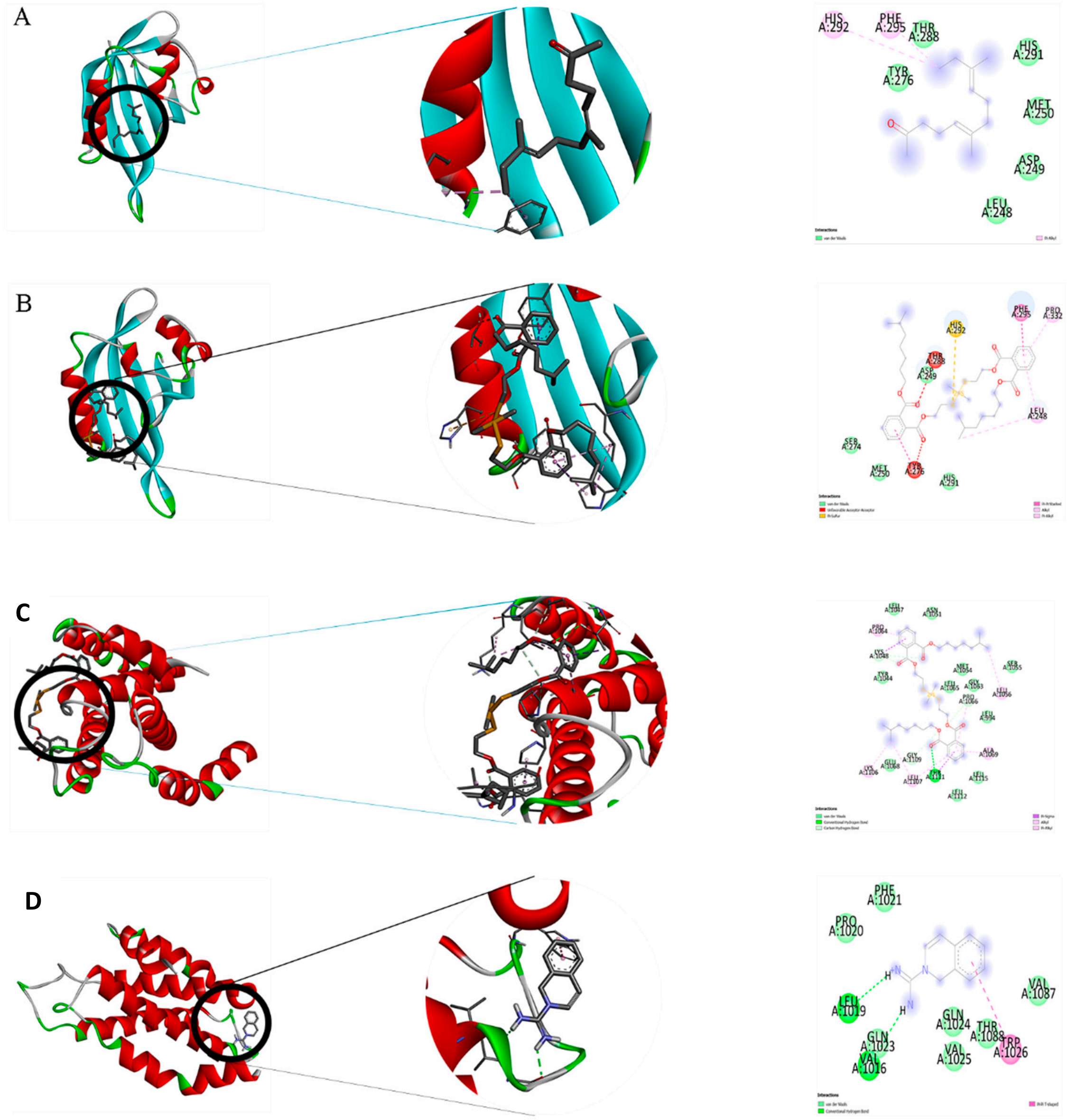

Figure S6.
The 3D plot of the binding sites and the 2D plot of interactions of HIF-1α with A) 6,10-Dimethyldodeca-5,9-dien-2-one, and B) 1, 2-benzene dicarboxylic acid, diisooctyl ester. The 3D plot of the binding sites and the 2D plot of interactions of TERT with C) 1, 2-benzene dicarboxylic acid, diisooctyl ester, and D) 3,4-Dihydro-1h-isoquinoline-2-carboxamidine hydrochloride.

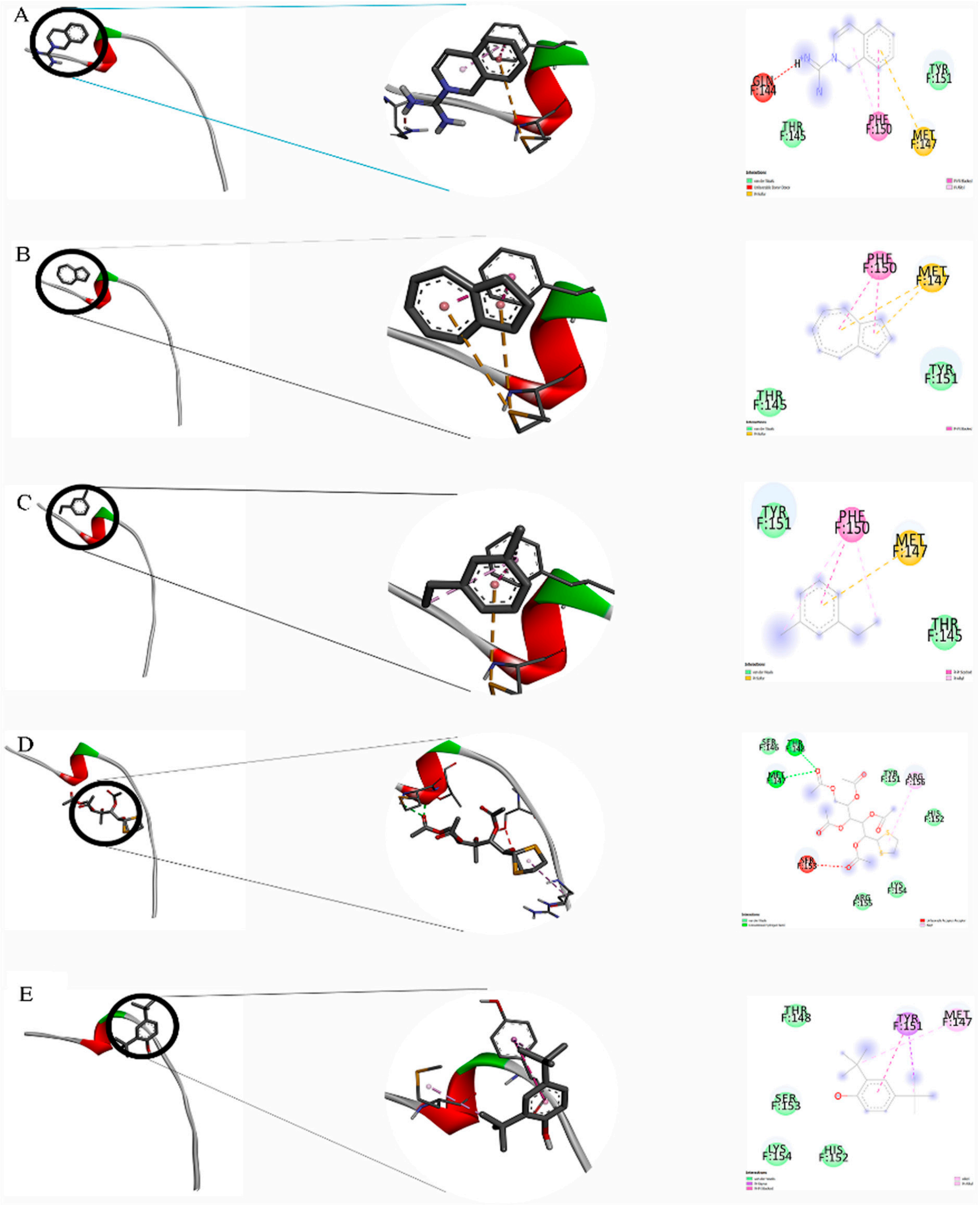

Figure S7.
The 3D plot of the binding sites and the 2D plot of interactions of p21 with A) 3,4-Dihydro-1h-isoquinoline-2-carboxamidine hydrochloride, B) Azulene, C) Benzene 1-ethyl-3-methyl-, D) D-glucose, cyclic ethylene mercaptan, pentaacetate, and E) Phenol,2,4-bis(1,1dimethylethyl)-.

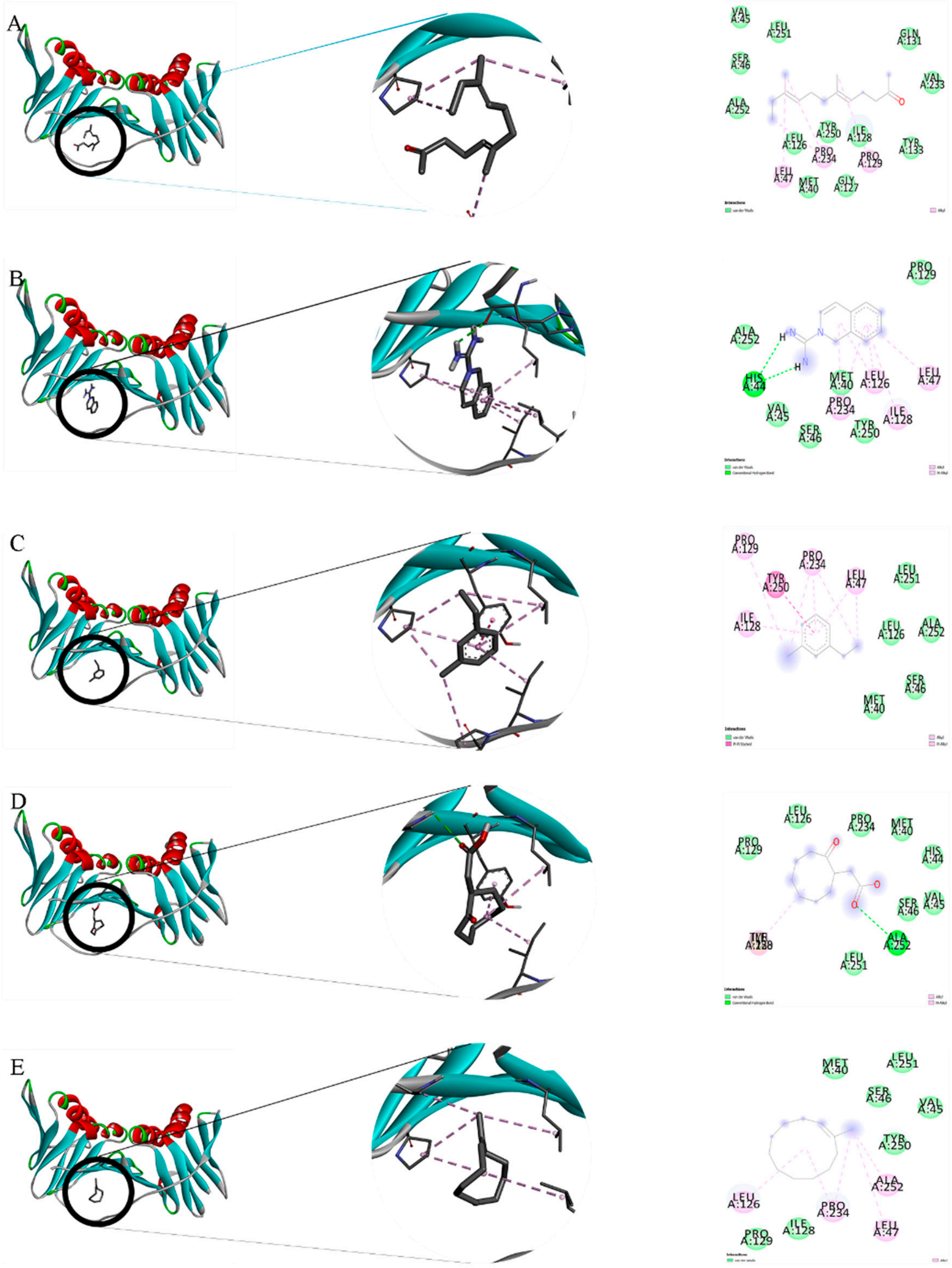

Figure S8.
The 3D plot of the binding sites and the 2D plot of interactions of PCNA with A) (5E,9E)-6,10-Dimethyldodeca-5,9-dien-2-one, B) 3,4-Dihydro-1h-isoquinoline-2-carboxamidine hydrochloride, C) Benzene 1-ethyl-3-methyl-, D) Cyclooctaneacetic acid, 2-oxo-, and E) Cycloundecene,1-methyl-.

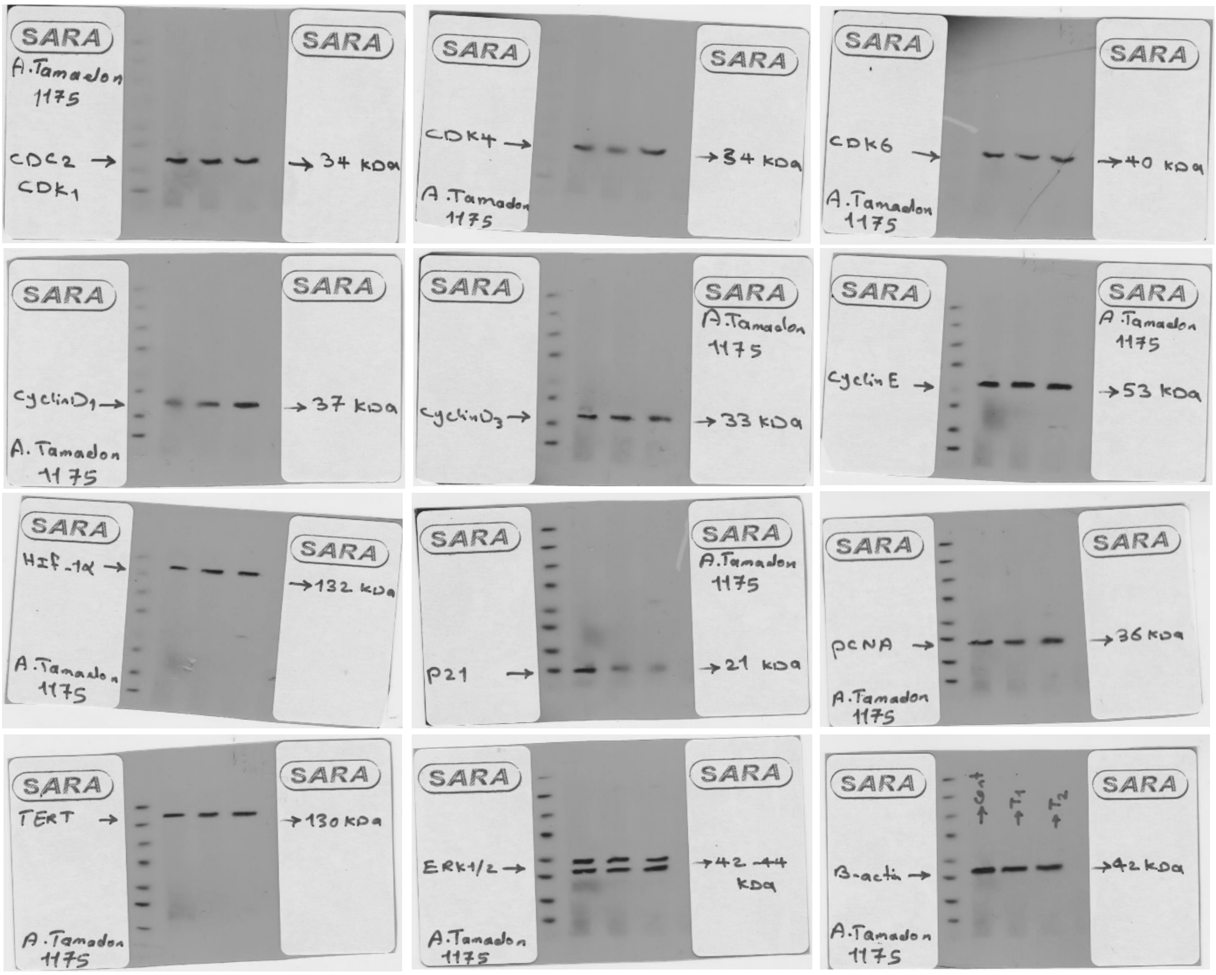

Figure S9.
The original gels of western blot analysis

References

1. Chen, S.; Liu, J.; Gong, H.; Yang, D. Identification and antibacterial activity of secondary metabolites from Taxus endophytic fungus. *Chin. J. Biotechnol.* **2009**, *25*, 368-374.
2. Hakozaki, T.; Laughlin, T.; Zhao, S.; Wang, J.; Deng, D.; Jewell-Motz, E.; Elstun, L. A regulator of ubiquitin-proteasome activity, 2-hexyldecanol, suppresses melanin synthesis and the appearance of facial hyperpigmented spots. *Br. J. Dermatol.* **2013**, *169 Suppl 2*, 39-44, doi:10.1111/bjd.12364.
3. Shirani, M.; Samimi, A.; Kalantari, H.; Madani, M.; Kord Zanganeh, A. Chemical composition and antifungal effect of hydroalcoholic extract of *Allium tripedale* (Tvautv.) against *Candida* species. *Curr. Med. Mycol.* **2017**, *3*, 6-12, doi:10.18869/acadpub.cmm.3.1.6.
4. Figueiredo, C.R.; Matsuo, A.L.; Massaoka, M.H.; Girola, N.; Azevedo, R.A.; Rabaca, A.N.; Farias, C.F.; Pereira, F.V.; Matias, N.S.; Silva, L.P.; et al. Antitumor activity of kielmeyera coriacea leaf constituents in experimental melanoma, tested *in vitro* and *in vivo* in syngeneic mice. *Adv. Pharm. Bull.* **2014**, *4*, 429-436, doi:10.5681/apb.2014.063.
5. Vambe, M.; Naidoo, D.; Aremu, A.O.; Finnie, J.F.; Van Staden, J. Bioassay-guided purification, GC-MS characterization and quantification of phyto-components in an antibacterial extract of *Searsia lancea* leaves. *Nat. Prod. Res.* **2021**, *35*, 4658-4662, doi:10.1080/14786419.2019.1700251.

6. Idle, J.R.; Mahgoub, A.; Angelo, M.M.; Dring, L.G.; Lancaster, R.; Smith, R.L. The metabolism of [14C]-debrisoquine in man. *Br. J. Clin. Pharmacol.* **1979**, *7*, 257-266, doi:10.1111/j.1365-2125.1979.tb00930.x.
7. Lim, C.S.; Wong, W.F.; Rosli, R.; Ng, K.P.; Seow, H.F.; Chong, P.P. 2-dodecanol (decyl methyl carbinol) inhibits hyphal formation and SIR2 expression in *C. albicans*. *J. Basic Microbiol.* **2009**, *49*, 579-583, doi:10.1002/jobm.200900035.
8. Rajendran, B.K.; Xavier Suresh, M.; Bhaskaran, S.P.; Harshitha, Y.; Gaur, U.; Kwok, H.F. Pharmacoinformatic approach to explore the antidote potential of phytochemicals on bungarotoxin from Indian krait, *Bungarus caeruleus*. *Comput. Struct. Biotechnol. J.* **2018**, *16*, 450-461, doi:10.1016/j.csbj.2018.10.005.
9. Witkowska-Banaszczak, E.; Długaszewska, J. Essential oils and hydrophilic extracts from the leaves and flowers of *Succisa pratensis* Moench. and their biological activity. *J. Pharm. Pharmacol.* **2017**, *69*, 1531-1539.
10. Bolchoz, L.J.; Budinsky, R.A.; McMillan, D.C.; Jollow, D.J. Primaquine-induced hemolytic anemia: formation and hemotoxicity of the arylhydroxylamine metabolite 6-methoxy-8-hydroxylaminoquinoline. *J. Pharmacol. Exp. Ther.* **2001**, *297*, 509-515.
11. Terekhova, E.A.; Stepicheva, N.A.; Pshenichnikova, A.B.; Shvets, V.I. Stearic acid methyl ether: a new extracellular metabolite of the obligate methylotrophic bacterium Methylophilus quaylei. *Prikl. Biokhim. Mikrobiol.* **2010**, *46*, 180-186.
12. Othman, A.R.; Abdullah, N.; Ahmad, S.; Ismail, I.S.; Zakaria, M.P. Elucidation of in-vitro anti-inflammatory bioactive compounds isolated from *Jatropha curcas* L. plant root. *BMC Complement. Altern. Med.* **2015**, *15*, 11, doi:10.1186/s12906-015-0528-4.
13. Chen, P.Y.; Wu, C.Y.; Clemons, G.A.; Citadin, C.T.; Couto, E.S.A.; Possoit, H.E.; Azizbayeva, R.; Forren, N.E.; Liu, C.H.; Rao, K.N.S.; et al. Stearic acid methyl ester affords neuroprotection and improves functional outcomes after cardiac arrest. *Prostaglandins Leukot. Essent. Fatty Acids* **2020**, *159*, 102138, doi:10.1016/j.plefa.2020.102138.
14. Doukas, P.H.; Speaker, T.J.; Thompson, R.S. Azulene analogs of pharmacological agents III: acute toxicity and local anesthetic activity of azulylamides and azulenecarboxamides. *J. Pharm. Sci.* **1975**, *64*, 158-161, doi:10.1002/jps.2600640137.
15. Chen, C.H.; Lee, O.; Yao, C.N.; Chuang, M.Y.; Chang, Y.L.; Chang, M.H.; Wen, Y.F.; Yang, W.H.; Ko, C.H.; Chou, N.T.; et al. Novel azulene-based derivatives as potent multi-receptor tyrosine kinase inhibitors. *Bioorg. Med. Chem. Lett.* **2010**, *20*, 6129-6132, doi:10.1016/j.bmcl.2010.08.025.
16. Panagiotopoulos, A.; Tseliou, M.; Karakasiliotis, I.; Kotzampasi, D.M.; Daskalakis, V.; Kesesidis, N.; Notas, G.; Lionis, C.; Kampa, M.; Pirintsos, S.; et al. p-cymene impairs SARS-CoV-2 and influenza A (H1N1) viral replication: *In silico* predicted interaction with SARS-CoV-2 nucleocapsid protein and H1N1 nucleoprotein. *Pharmacol. Res. Perspect.* **2021**, *9*, e00798, doi:10.1002/prp2.798.
17. Formiga, R.O.; Alves Junior, E.B.; Vasconcelos, R.C.; Araujo, A.A.; de Carvalho, T.G.; de Araujo Junior, R.F.; Guerra, G.B.C.; Vieira, G.C.; de Oliveira, K.M.; Diniz, M.; et al. Effect of p-cymene and rosmarinic acid on gastric ulcer healing - Involvement of multiple endogenous curative mechanisms. *Phytomedicine.* **2021**, *86*, 153497, doi:10.1016/j.phymed.2021.153497.
18. Hassan, S.M.H.; Ray, P.; Hossain, R.; Islam, M.T.; Salehi, B.; Martins, N.; Sharifi-Rad, J.; Amarowicz, R. p-Cymene metallo-derivatives: An overview on anticancer activity. *Cell Mol. Biol.* **2020**, *66*, 28-32.
19. Aoki, S.; Ohta, K.; Matsumoto, K.; Sakai, H.; Abe, M.; Miura, M.; Sugawara, F.; Sakaguchi, K. An emulsion of sulfoquinovosylacylglycerol with long-chain alkanes

increases its permeability to tumor cells. *J. Membr. Biol.* **2006**, *213*, 11-18, doi:10.1007/s00232-006-0054-x.
20. Dhouibi, R.; Moalla, D.; Ksouda, K.; Ben Salem, M.; Hammami, S.; Sahnoun, Z.; Zeghal, K.M.; Affes, H. Screening of analgesic activity of Tunisian *Urtica dioica* and analysis of its major bioactive compounds by GCMS. *Arch. Physiol. Biochem.* **2018**, *124*, 335-343, doi:10.1080/13813455.2017.1402352.
21. Thabet, A.A.; Youssef, F.S.; El-Shazly, M.; AN, B.S. GC-MS and GC-FID analyses of the volatile constituents of *Brachychiton rupestris* and Brachychiton discolor, their biological activities and their differentiation using multivariate data analysis. *Nat. Prod. Res.* **2020**, *34*, 590-594, doi:10.1080/14786419.2018.1490908.
22. Herman, S.; Kny, A.; Schorn, C.; Pfatschbacher, J.; Niederreiter, B.; Herrmann, M.; Holmdahl, R.; Steiner, G.; Hoffmann, M.H. Cell death and cytokine production induced by autoimmunogenic hydrocarbon oils. *Autoimmunity* **2012**, *45*, 602-611, doi:10.3109/08916934.2012.719948.
23. Yadalam, P.K.; Varatharajan, K.; Rajapandian, K.; Chopra, P.; Arumuganainar, D.; Nagarathnam, T.; Sohn, H.; Madhavan, T. Antiviral essential oil components against SARS-CoV-2 in pre-procedural mouth rinses for dental settings during COVID-19: A computational study. *Front. Chem.* **2021**, *9*, 642026, doi:10.3389/fchem.2021.642026.
24. El Yaagoubi, M.; Ortiz, S.; Mechqoq, H.; Cavaleiro, C.; Lecso-Bornet, M.; Rodrigues, M.J.; Custodio, L.; El Mousadik, A.; Grougnet, R.; El Aouad, N.; et al. Chemical composition, antibacterial screening and cytotoxic activity of *Chiliadenus antiatlanticus* (Emb. & Maire) Gómiz (Asteraceae) essential oil. *Chem. Biodivers.* **2021**, *18*, e2100115, doi:10.1002/cbdv.202100115.
25. George, J.D.; Price, C.J.; Marr, M.C.; Myers, C.B.; Schwetz, B.A.; Heindel, J.J. Evaluation of the developmental toxicity of methacrylamide and N,N'-methylenebisacrylamide in Swiss mice. *Toxicol. Sci.* **1998**, *46*, 124-133, doi:10.1006/toxs.1998.2506.
26. Mozaffarian, D.; Cao, H.; King, I.B.; Lemaitre, R.N.; Song, X.; Siscovick, D.S.; Hotamisligil, G.S. Circulating palmitoleic acid and risk of metabolic abnormalities and new-onset diabetes. *Am. J. Clin. Nutr.* **2010**, *92*, 1350-1358, doi:10.3945/ajcn.110.003970.
27. Zhang, L.; Lv, J.; Chen, C.; Wang, X. Roles of acyl-CoA synthetase long-chain family member 5 and colony stimulating factor 2 in inhibition of palmitic or stearic acids in lung cancer cell proliferation and metabolism. *Cell Biol. Toxicol.* **2021**, *37*, 15-34, doi:10.1007/s10565-020-09520-w.
28. Bi, C.; Zhang, T.; Li, Y.; Zhao, H.; Zhang, P.; Wang, Y.; Xu, Y.; Gu, K.; Liu, Y.; Yu, J.; et al. A proteomics- and metabolomics-based study revealed that disorder of palmitic acid metabolism by aconitine induces cardiac injury. *Chem. Res. Toxicol.* **2020**, *33*, 3031-3040, doi:10.1021/acs.chemrestox.0c00372.
29. Sanchez-Alegria, K.; Bastian-Eugenio, C.E.; Vaca, L.; Arias, C. Palmitic acid induces insulin resistance by a mechanism associated with energy metabolism and calcium entry in neuronal cells. *FASEB J.* **2021**, *35*, e21712, doi:10.1096/fj.202100243R.
30. Galindo-Hernandez, O.; Leija-Montoya, A.G.; Romero-Garcia, T.; Vazquez-Jimenez, J.G. Palmitic acid decreases cell migration by increasing RGS2 expression and decreasing SERCA expression. *Genet. Mol. Biol.* **2021**, *44*, e20200279, doi:10.1590/1678-4685-GMB-2020-0279.
31. Korbecki, J.; Bajdak-Rusinek, K. The effect of palmitic acid on inflammatory response in macrophages: An overview of molecular mechanisms. *Inflamm. Res.* **2019**, *68*, 915-932, doi:10.1007/s00011-019-01273-5.

32. Marrez, D.A.; Naguib, M.M.; Sultan, Y.Y.; Higazy, A.M. Antimicrobial and anticancer activities of *Scenedesmus obliquus* metabolites. *Heliyon* **2019**, *5*, e01404, doi:10.1016/j.heliyon.2019.e01404.
33. Rajkumar, S.; Jebanesan, A. Mosquitocidal activities of octacosane from *Moschosma polystachyum* Linn (lamiaceae). *J. Ethnopharmacol.* **2004**, *90*, 87-89, doi:10.1016/j.jep.2003.09.030.
34. Jing, L.L.; He, L.; Fan, P.C.; Jia, Z.P.; Ma, H.P. Chemical constituents with anti-hypoxia activity from *Saussurea involucrata*. *J. Chin. Med. Mat.* **2015**, *38*, 89-92.
35. Figueiredo, C.R.; Matsuo, A.L.; Pereira, F.V.; Rabaca, A.N.; Farias, C.F.; Girola, N.; Massaoka, M.H.; Azevedo, R.A.; Scutti, J.A.; Arruda, D.C.; et al. *Pyrostegia venusta* heptane extract containing saturated aliphatic hydrocarbons induces apoptosis on B16F10-Nex2 melanoma cells and displays antitumor activity in vivo. *Pharmacogn. Mag.* **2014**, *10*, S363-S376, doi:10.4103/0973-1296.133284.
36. Tátrai, E.; Ungváry, G.; Cseh, I.R.; Mányai, S.; Szeberényi, S.; Molnár, J.; Morvai, V. The effect of long-term inhalation of ortho-xylene on the liver. In Proceedings of the Industrial and Environmental Xenobiotics, Berlin, Heidelberg, 1981; pp. 293-300.
37. Sarma, S.N.; Kim, Y.J.; Song, M.; Ryu, J.C. Induction of apoptosis in human leukemia cells through the production of reactive oxygen species and activation of HMOX1 and Noxa by benzene, toluene, and o-xylene. *Toxicology* **2011**, *280*, 109-117, doi:10.1016/j.tox.2010.11.017.
38. Varsha, K.K.; Devendra, L.; Shilpa, G.; Priya, S.; Pandey, A.; Nampoothiri, K.M. 2,4-di-tert-butyl phenol as the antifungal, antioxidant bioactive purified from a newly isolated *Lactococcus* sp. *Int. J. Food Microbiol.* **2015**, *211*, 44-50, doi:10.1016/j.ijfoodmicro.2015.06.025.
39. Zhao, F.; Wang, P.; Lucardi, R.D.; Su, Z.; Li, S. Natural sources and bioactivities of 2,4-Di-Tert-butylphenol and its analogs. *Toxins* **2020**, *12*, 35, doi:10.3390/toxins12010035.
40. Chathuranga, K.; Weerawardhana, A.; Dodantenna, N.; Ranathunga, L.; Cho, W.K.; Ma, J.Y.; Lee, J.S. Inhibitory effect of *Sargassum fusiforme* and its components on replication of respiratory syncytial virus in vitro and in vivo. *Viruses* **2021**, *13*, 548, doi:10.3390/v13040548.
41. Paudel, M.R.; Chand, M.B.; Pant, B.; Pant, B. Assessment of antioxidant and cytotoxic activities of extracts of *Dendrobium crepidatum*. *Biomolecules* **2019**, *9*, 478, doi:10.3390/biom9090478.
42. Uddin, S.J.; Grice, D.; Tiralongo, E. Evaluation of cytotoxic activity of patriscabratine, tetracosane and various flavonoids isolated from the Bangladeshi medicinal plant *Acrostichum aureum*. *Pharm. Biol.* **2012**, *50*, 1276-1280.
43. Muhammad, F.; Monteiro-Riviere, N.A.; Riviere, J.E. Comparative *in vivo* toxicity of topical JP-8 jet fuel and its individual hydrocarbon components: identification of tridecane and tetradecane as key constituents responsible for dermal irritation. *Toxicol. Pathol.* **2005**, *33*, 258-266, doi:10.1080/01926230590908222.
44. Sharma, R.; Locke, B.R. Jet fuel toxicity: skin damage measured by 900-MHz MRI skin microscopy and visualization by 3D MR image processing. *Magn. Reson. Imaging* **2010**, *28*, 1030-1048, doi:10.1016/j.mri.2010.03.045.
45. Zazula, R.; Moravec, M.; Pehal, F.; Nejtek, T.; Protus, M.; Muller, M. Myristic Acid Serum Levels and Their Significance for Diagnosis of Systemic Inflammatory Response, Sepsis, and Bacteraemia. *J. Pers. Med.* **2021**, *11*, 306, doi:10.3390/jpm11040306.
46. Khalil, A.S.M.; Giribabu, N.; Yelumalai, S.; Shahzad, H.; Kilari, E.K.; Salleh, N. Myristic acid defends against testicular oxidative stress, inflammation, apoptosis:

Restoration of spermatogenesis, steroidogenesis in diabetic rats. *Life Sci.* **2021**, *278*, 119605, doi:10.1016/j.lfs.2021.119605.
47. Kim, Y.G.; Lee, J.H.; Park, S.; Kim, S.; Lee, J. Inhibition of polymicrobial biofilm formation by saw palmetto oil, lauric acid and myristic acid. *Microb. Biotechnol.* **2022**, *15*, 590-602, doi:10.1111/1751-7915.13864.
48. Singh, S.; Singh, J. Percutaneous absorption, biophysical, and macroscopic barrier properties of porcine skin exposed to major components of JP-8 jet fuel. *Environ. Toxicol. Pharmacol.* **2003**, *14*, 77-85, doi:10.1016/S1382-6689(03)00028-0.
49. Singh, S.; Singh, J. Dermal toxicity and microscopic alterations by JP-8 jet fuel components in vivo in rabbit. *Environ. Toxicol. Pharmacol.* **2004**, *16*, 153-161, doi:10.1016/j.etap.2003.12.001.
50. Choi, D.; Kang, W.; Park, T. Anti-allergic and anti-inflammatory effects of undecane on mast cells and keratinocytes. *Molecules* **2020**, *25*, 1554, doi:10.3390/molecules25071554.
51. Nagella, P.; Ahmad, A.; Kim, S.J.; Chung, I.M. Chemical composition, antioxidant activity and larvicidal effects of essential oil from leaves of *Apium graveolens*. *Immunopharmacol. Immunotoxicol.* **2012**, *34*, 205-209, doi:10.3109/08923973.2011.592534.
